# Project-level causal inference of a minimum viable scale threshold and adoption band in CCUS-enabled hydrogen

**DOI:** 10.1016/j.isci.2026.117236

**Published:** 2026-08-25

**Authors:** Joseph Junior Nkou Nkou, Olusola Bamisile, Chukwuebuka J. Ejiyi, Anto Jonathan Leoba, Qi Huang, Cai Dongsheng

**Affiliations:** 1 College of Nuclear Technology and Automation Engineering, Chengdu University of Technology, Chengdu, Sichuan P.R 610059, China; 2 Sichuan Engineering Technology Research Center for Industrial Internet Intelligent Monitoring and Application, Chengdu University of Technology, Chengdu, Sichuan P.R 610059, China; 3 Division of Energy, Environment, and Society, University of Dundee, Dundee, UK

**Keywords:** carbon capture and storage, CCS, hydrogen production capacity, causal inference, heterogeneous treatment effects, energy policy

## Abstract

In this study, we analyze whether adopting carbon capture, utilization, and storage (CCUS) causally raises the deployable capacity of hydrogen projects, and under what scale and infrastructure conditions any such effect emerges. Analyzing 2,437 announced global hydrogen projects (182 with CCUS, 2,255 without) from the IEA Hydrogen Production Projects Database 2025, we apply a project-level multi-estimator causal-inference stack—augmented inverse probability weighting, propensity-score matching, X-Learner and DR-Learner—with binary-segmentation changepoint detection, Rosenbaum-bound and *E*-value sensitivity, permutation placebos, region-exclusion tests, and independent exposure validation against the IEA CCUS Projects Database. CCUS behaves as a scale- and context-gated amplifier: capacity uplift concentrates in the 500- 1,000 MWel adoption band with a +1.45× within-stratum effect above the minimum-viable ≥500-MWel scale threshold (Rosenbaum Γ = 3.7; *E*-value = 12.4) and no detectable effect at smaller scales. The finding reframes CCUS-hydrogen policy design, integrated-assessment modeling, and project finance around scale-conditional CO_2_ transport-and-storage and policy prerequisites.

## Highlights

• CCUS raises hydrogen project capacity only above a 500-MWel threshold
• 500-1,000-MWel adoption band: +1.45× within-stratum uplift above the threshold
• Effect robust to unmeasured confounding (Rosenbaum Γ = 3.7; *E*-value = 12.4)
• CCUS is a scale- and context-gated amplifier, not a universal lever

## Graphical abstract

**Figure:**
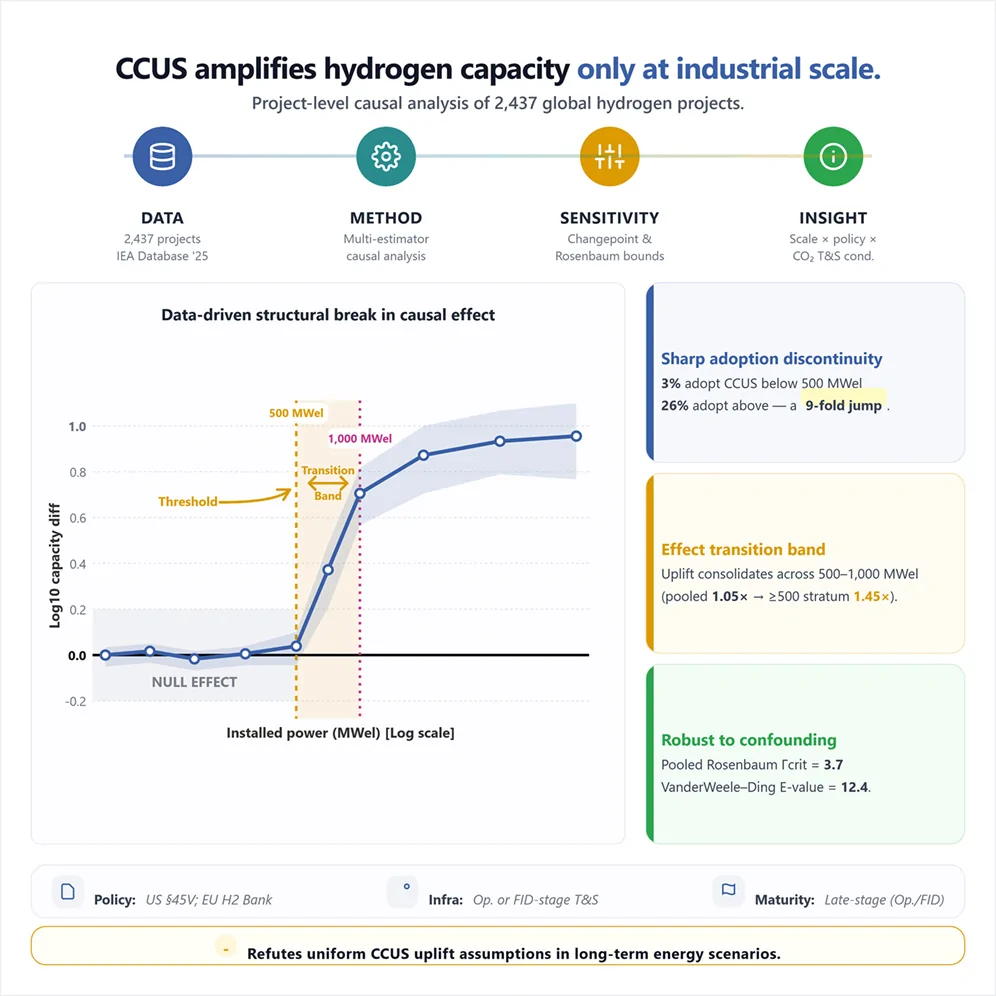

## Teaser

Applied sciences; Energy storage

## Introduction

Hydrogen is increasingly considered as a structural pillar of deep decarbonization rather than a niche energy carrier, with proposed roles spanning steel, chemicals, refining, power balancing, and heavy transport. Recent reviews of industrial decarbonization argue that hydrogen can substitute fossil feedstocks and high-temperature fuels across multiple value chains, provided that large volumes of low-carbon supply can be mobilized and integrated into existing infrastructures. At the same time, global project pipelines and policy roadmaps suggest a rapid expansion of hydrogen capacity but highlight substantial uncertainty over how much of the announced pipeline will translate into realized, climate-aligned infrastructure. In particular, the emerging distinction between ambition and implementation—the “green hydrogen gap”—raises questions about which technological and infrastructural choices most effectively scale deployable hydrogen capacity. A recent global hydrogen budget reconstruction emphasizes that uncertainty in current emission factors and project-level deployment data is a major constraint on credible mitigation accounting.^1^ This empirical gap is the gap our project-level causal design directly targets: rather than aggregating scenario-derived intensities, we estimate within-stratum effects from observed project records, with the trim-band and Rosenbaum sensitivity required to assess how robust any conditional conclusion is.^2^^,^^3^^,^^4^

A growing body of life cycle assessment (LCA) and energy-systems modeling work shows that the climate performance of hydrogen is strongly conditioned by its system context rather than technology alone. Prospective global LCAs of green hydrogen quantify how upstream electricity mixes, siting decisions, and transport pathways can shift well-to-gate emissions by factors of two or more.^5^ Coupled LCA-integrated assessment studies further demonstrate that temporal resolution, regional granularity, and scenario assumptions materially affect whether hydrogen pathways deliver genuine mitigation or merely relocate emissions.^6^^,^^7^ Together, these findings imply that evaluating hydrogen solely at the level of process efficiencies is insufficient: robust assessment must account for where and how projects are embedded in power systems, infrastructures, and carbon management regimes.

Within this broader picture, “blue” hydrogen—fossil-based hydrogen coupled to carbon capture, utilization, and storage (CCUS)—has emerged as both a promising and controversial option. Process engineering and systems analyses indicate that steam methane reforming (SMR), autothermal reforming (ATR), or gasification with high capture rates could, in principle, deliver low life cycle emissions if upstream methane leakage is controlled and storage is secure. However, other work finds that realistic methane leakage and incomplete capture can leave blue hydrogen with life cycle emissions comparable to or worse than direct combustion of natural gas. Subsequent analyses demonstrate that under stringent capture rates and tight methane control, blue hydrogen can approach green hydrogen’s climate performance but only under specific boundary conditions. The emerging consensus is therefore not that blue hydrogen is inherently incompatible with net-zero targets, but that its climate value is conditioned on storage performance, upstream gas management, and durable storage.^8^^,^^9^^,^^10^^,^^11^^,^^12^

Economic and geospatial factors add further layers of conditionality. Comparative techno-economic assessments show that the cost competitiveness of blue versus green hydrogen is highly sensitive to gas prices, carbon prices, electrolysis costs, and load factors. Global and regional studies of existing and planned hydrogen projects reveal strong spatial variation in both cost and emissions intensity, driven by renewable resource quality, electricity mix, and CO_2_ storage availability. In Europe and other industrial regions, qualitative and mixed-methods analyses of industrial clusters emphasize that hydrogen and CCS tend to be most viable where they can be co-developed with shared infrastructure and strong policy support. These studies reinforce that hydrogen-CCUS pathways are not homogeneous; they are shaped by local resource endowments, regulatory regimes, and institutional capacity.^13^^,^^14^^,^^15^

Engineering analyses of CCUS underline why scale and infrastructure configuration are central. Large-scale capture, transport, and storage systems sustained multi-megaton CO_2_ flows to justify pipeline networks and reservoir development. Studies of hubs and clusters in Spain and elsewhere demonstrate that coordinated CCUS deployment across multiple emitters can substantially reduce unit costs compared with stand-alone projects, mainly by spreading fixed transport-and-storage (T&S) investments over larger captured volumes. European power-system and industry scenarios similarly find that CCUS becomes cost-effective only beyond certain plant sizes and cluster scales, where economies of scale in capture units and shared transport infrastructure can be realized. Recent work on CCUS clusters and hubs in China and at provincial scale in Anhui shows that optimized source-sink matching and cluster deployment can cut pipeline length and investment by 25–70, while still accommodating very large cumulative storage volumes. Contemporary CCUS reviews converge on the view that cluster-based, infrastructure-centric deployment is rapidly becoming the dominant paradigm for cost-effective CCUS.^16^^,^^17^^,^^18^^,^^19^^,^^20^

Despite this rich landscape of modeling, engineering analysis, and qualitative work on hydrogen and CCUS, there remains a notable gap in empirical, project-level causal evidence on how CCUS adoption affects deployable hydrogen capacity. Most studies either use stylized plants, prospective designs, or scenario ensembles in integrated assessment models and techno-economic frameworks. Even analyses that compile global project databases focus on aggregated mitigation potential or life cycle emissions on planned hydrogen projects, without explicitly estimating treatment effects of CCUS adoption at the project level. As a result, two critical questions remain underexplored: (1) does adding CCUS systematically increase the deployable capacity of hydrogen projects in practice and (2) are any such effects uniform across scales, technologies, maturity stages, and regional policy environments—or instead gated by thresholds and context?^5^^,^^8^^,^^13^

This paper addresses these questions by assembling and analyzing a global project-level dataset of 2,437 hydrogen projects with and without CCUS, derived from the IEA’s hydrogen project database. We integrate multivariate project profiling with machine-learning-assisted adjustment, overlap diagnostics, and multi-estimator causal inference to estimate conditional average treatment effects () of CCUS adoption on nameplate hydrogen capacity. Specifically, we test whether CCUS functions as a scale-dependent and context-moderated amplifier of deployable hydrogen capacity, rather than a universally beneficial add-on. We stratify effects by project scale (MWel), technology family, project maturity, and regional policy context, thereby aligning our empirical design with the engineering logic of CCUS hubs and industrial clusters.^16^^,^^19^

Our results show that the capacity association with CCUS is strongly scale-dependent and concentrated in industrial-scale projects. CCUS adoption is sharply discontinuous at 500 MWel; within-stratum effects concentrate in a 500–1,000 MWel transition band identified by data-driven changepoint analysis. Inference at smaller scales is statistically inconclusive due to a practical positivity violation, rather than a demonstration of absence. Effects are moderated by technology, project maturity, and policy environment with operational CO_2_ T&S access, and are robust to plausible unmeasured confounding. The IEA database contains no fossil-without-CCUS hydrogen projects, so within-fossil-family counterfactuals are not estimable; cross-technology contrasts are reported in their place.

By providing one of the first global project-level causal assessments of CCUS impacts on hydrogen capacity, this study contributes three main advances. First, it empirically identifies a minimum viable scale regime for CCUS-enabled hydrogen projects, complementing engineering analyses of hubs and clusters. Second, it demonstrates that CCUS acts as a conditional amplifier of deployable capacity, gated by technology, maturity, and policy context rather than operating as a uniform lever. Third, it generates directly actionable evidence for infrastructure planning, industrial-cluster strategy, and energy-systems modeling, helping to align hydrogen roadmaps and CCUS deployment with the scales and context where these technologies materially expand capacity rather than simply adding cost and complexity. These insights are particularly relevant for regional hub developers and modelers seeking to represent hydrogen-CCUS interactions in a way that is consistent with both engineering constraints and observed project behavior.^14^^,^^15^

## Results

### CCS adoption is concentrated in large-scale industrial projects

**Figure 1:**
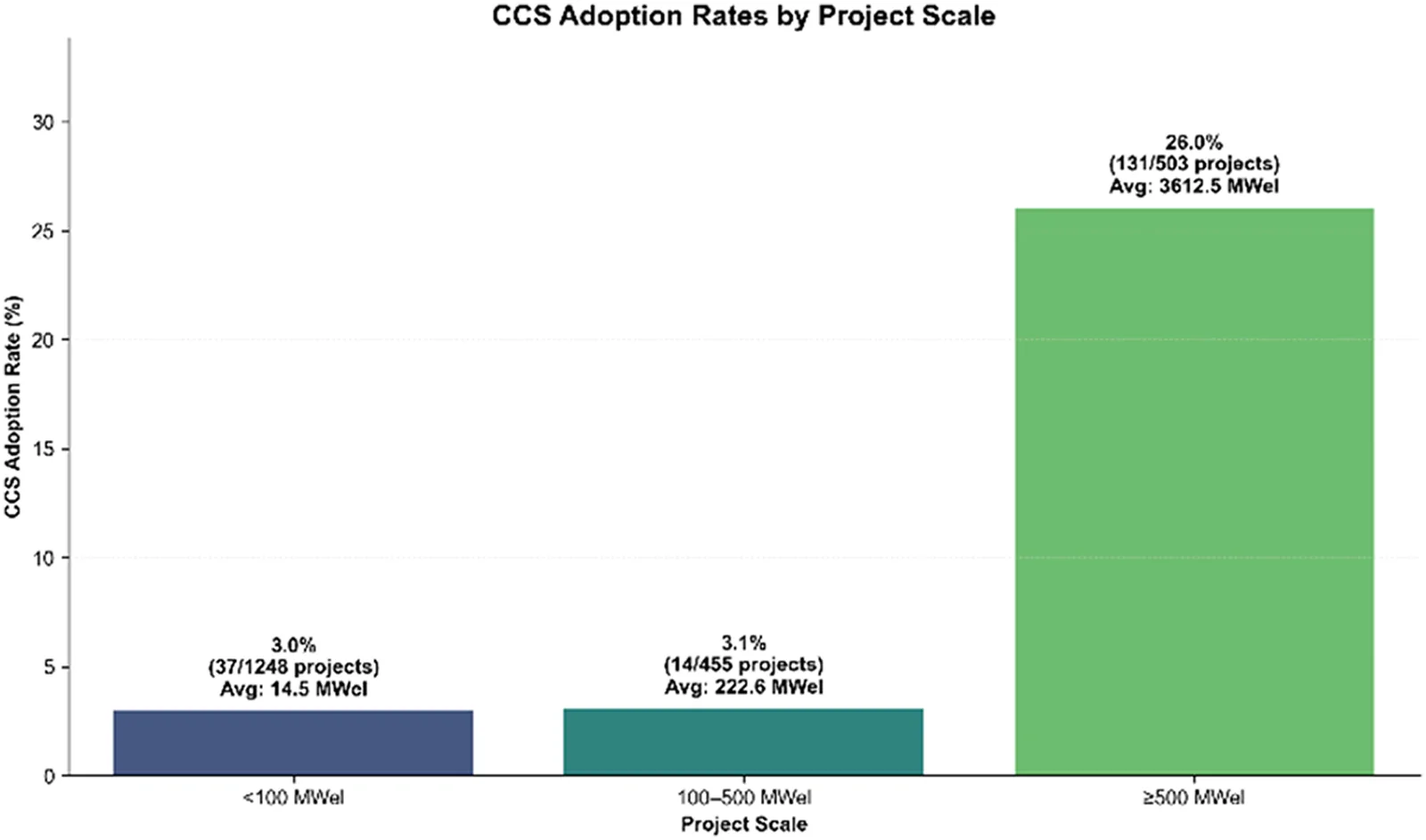
CCS adoption rates by project scale reveal a sharp threshold effect: negligible below 100 MWel, modest at 100–500 MWel, and significant integration only above 500 MWel From the 2,437 projects analyzed, only 182 integrate CCS. Adoption is nearly absent in small projects (<100 MWel, 3.0%) and remains marginal in mid-scale projects (100–500 MWel, 3.1%). Integration rises sharply at ≥500 MWel (26.0%), consistent with the hypothesis that CCS infrastructure imposes a minimum viable scale for economic feasibility. This threshold effect establishes scale-dependence as the primary structural condition for CCS adoption.

Our analysis of 2,437 projects (Table 1) revealed that only 182 integrate CCS (7.5%). Adoption was overwhelmingly dependent on project scale. Among the 2,206 projects reporting installed power, CCS was negligible at small scales (3.0% for <100 MWel (37/1,248)) and remained marginal in mid-scale projects (3.1% for 100-<500 MWel (14/455)). In contrast, CCS was adopted in 26.0% (131/503) of large-scale projects (for ≥500 MWel) (Figure 1).

**Table 1:** Dataset assembly and eligibility (projects)

Step	Description	N
S0	IEA database extract, de-duplicated and schema-validated	2,437
S1	predictive analysis set (non-missing outcome)	2,437
S2	causal estimation set (complete on exposure, outcome, confounders + common-support gates)	2,151

**Figure 2:**
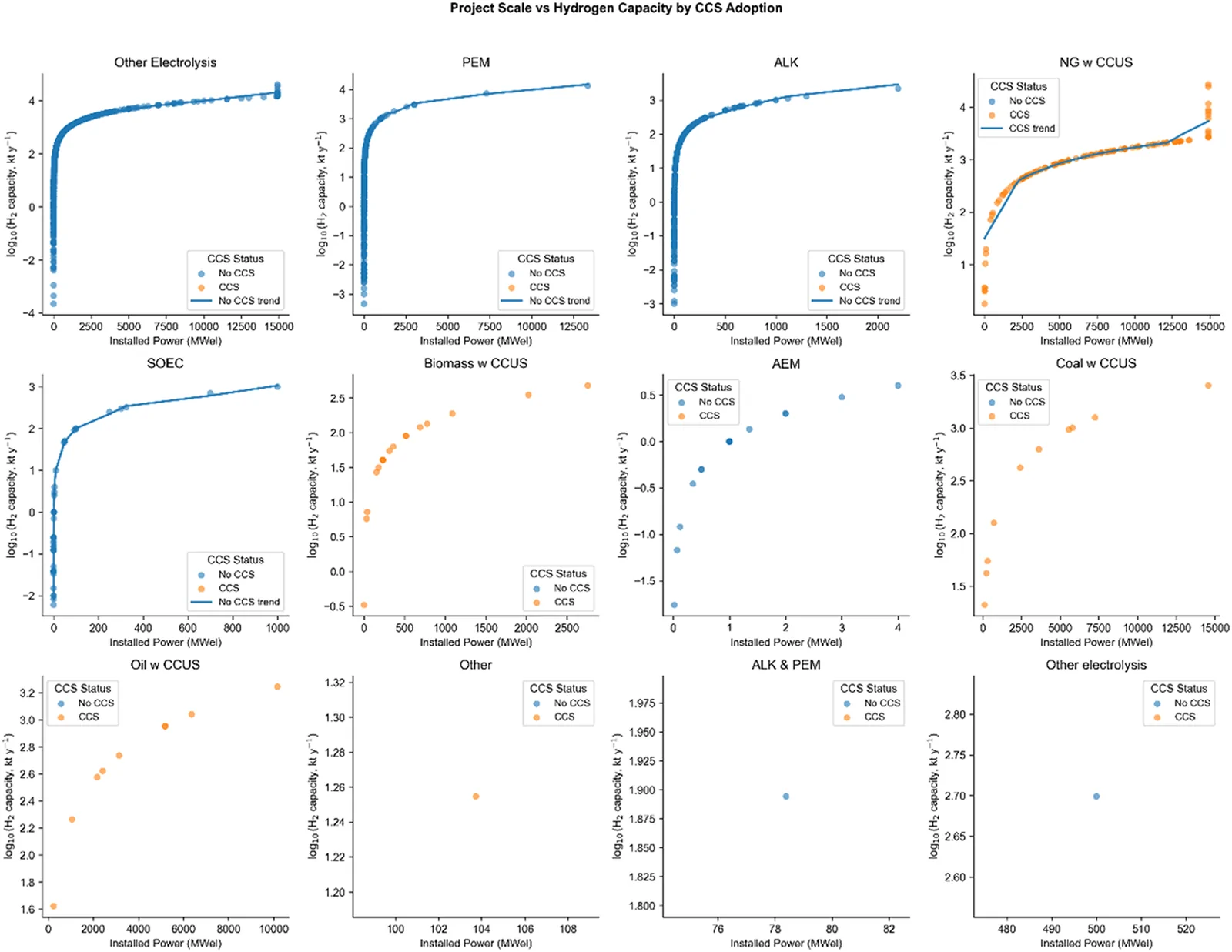
Technology-wise scatterplots with LOWESS smoothing illustrate CCS-enabled projects systematically skewed toward higher capacity bands, independent of electricity source Scatterplots of installed electrical capacity versus hydrogen output, smoothed with LOWESS regression, show CCS-enabled projects clustering at higher output bands regardless of electricity sourcing. Electrolytic projects appear widely distributed across capacities, confirming that CCS adoption is conditioned not by electricity inputs but by structural scale requirements. This reinforces the interpretation that CCS imposes additional capital and infrastructure constraints, in contrast to the relative scalability of electrolytic routes.

**Figure 3:**
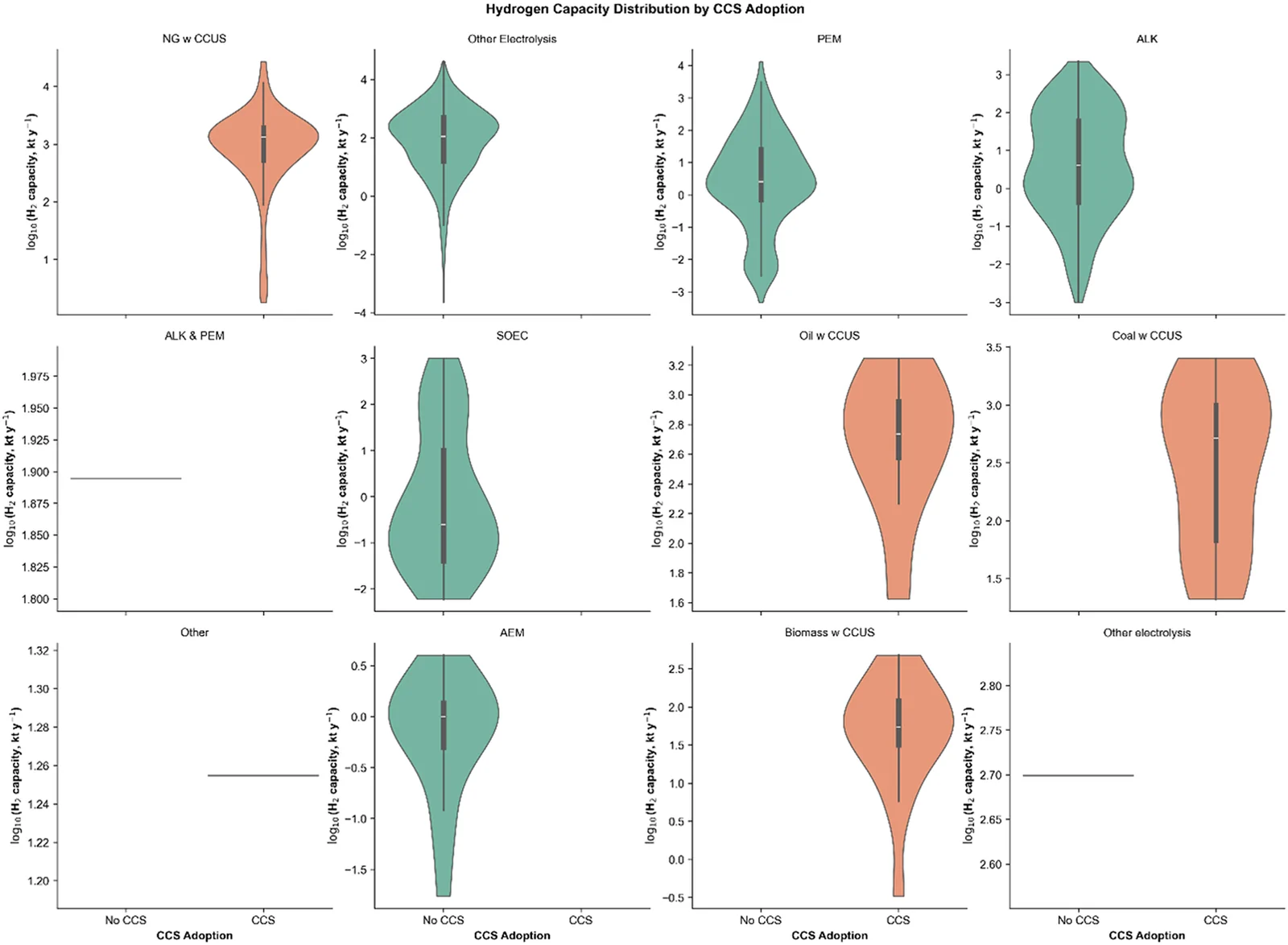
Violin plot of log-transformed hydrogen output shows CCS projects clustering at higher capacities with broad variance, contrasting with non-CCS projects concentrated at lower scales Violin plots compare the distribution of hydrogen output for CCS-enabled versus non-CCS projects, normalized on a log_10_ scale. CCS projects exhibit a higher median output but also broader variance, spanning pilot retrofits to large-scale hubs. Non-CCS projects dominate smaller-scale deployments, reflecting the prevalence of electrolysis at pilot and mid-scale levels. The broader dispersion among CCS projects highlights heterogeneity in their technological maturity and regional drivers. Horizontal line inside each violin = median; box = interquartile range (25th–75th percentile); whiskers = 1.5 × IQR; violin width proportional to kernel density.

**Figure 4:**
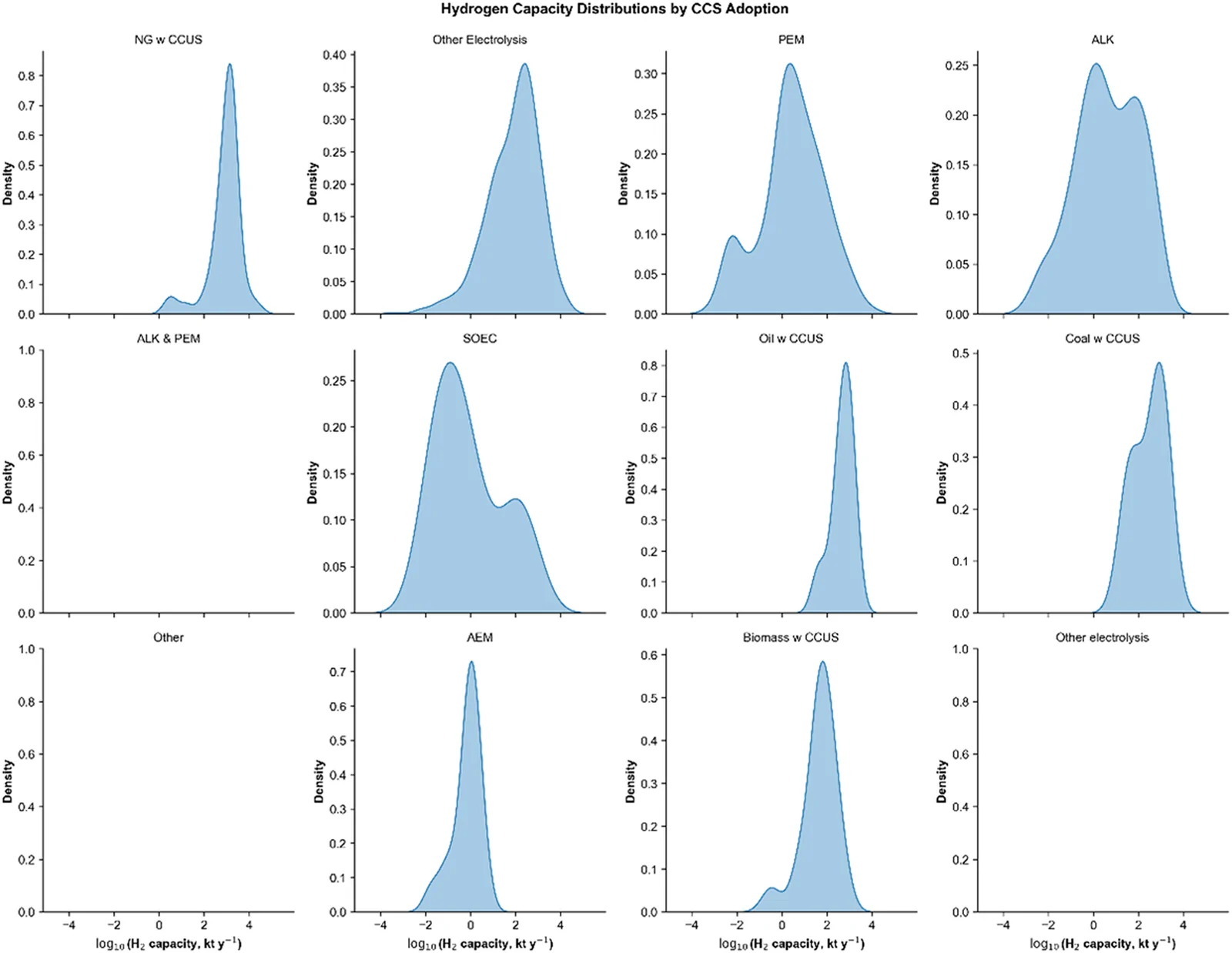
Kernel density estimates of hydrogen output confirm CCS projects disproportionately occupy the right-hand tail of the capacity distribution, while non-CCS projects dominate smaller scales Kernel density curves for annual hydrogen production capacity show distinct profiles: non-CCS projects are concentrated at lower outputs, with a bimodal pattern indicative of pilot- and medium-scale deployments, while CCS-enabled projects cluster near 10^2^ kt H_2_/year. The right-shifted distribution of CCS projects reflects the concentration of adoption in large-scale industrial plants, reinforcing the scale-threshold findings of Figure 1.

**Figure 5:**
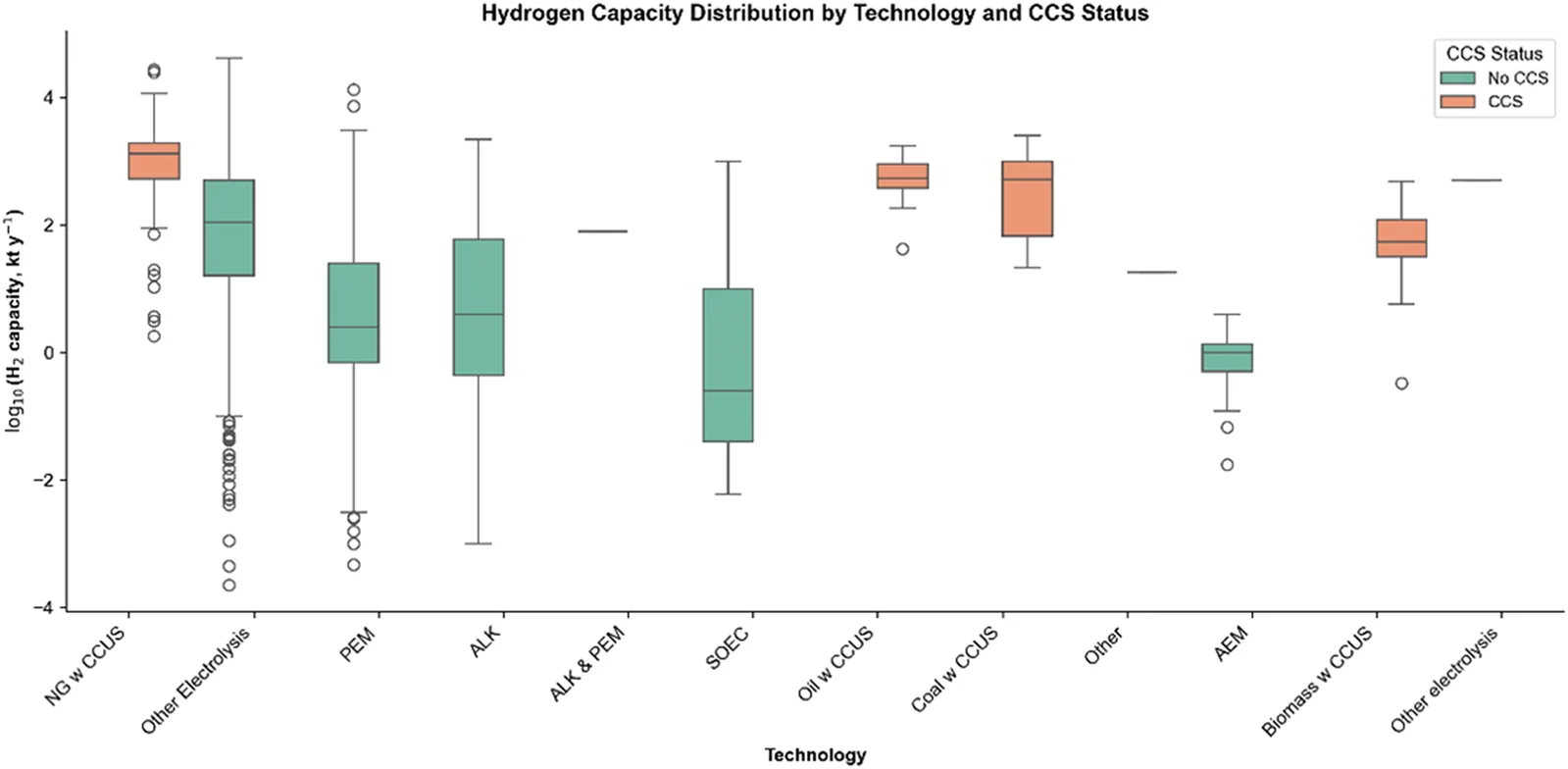
Technology-specific boxplots reveal fossil-based CCS pathways (coal, gas, biomass) cluster at larger scales, while electrolysis pathways remain more widely distributed but median-bounded below CCS projects Boxplots stratified by technology pathway show that coal gasification with CCS, ATR+CCUS, and SMR+CCUS dominate at larger scales, often exceeding 500 MWel. Electrolysis technologies (ALK, PEM, SOEC, AEM) display broader deployment across scales but with lower medians compared to CCS fossil pathways. The heterogeneity within CCS-enabled fossil technologies suggests pathway-specific cost and infrastructure determinants, while electrolysis maintains flexibility across small and medium projects. Box = interquartile range (25th–75th percentile); central line = median; whiskers = 1.5 × IQR; individual points = outliers.

This scaling pattern was consistent across technology types. Fossil-based projects were clustered at the high end of the capacity range, while electrolysis projects were distributed across all scales but with a lower median capacity (Figure 2). The distribution of project capacities for CCS and non-CCS projects was fundamentally different, with CCS projects strongly shifted toward higher outputs (Figures 3, 4, and 5).

**Figure 6:**
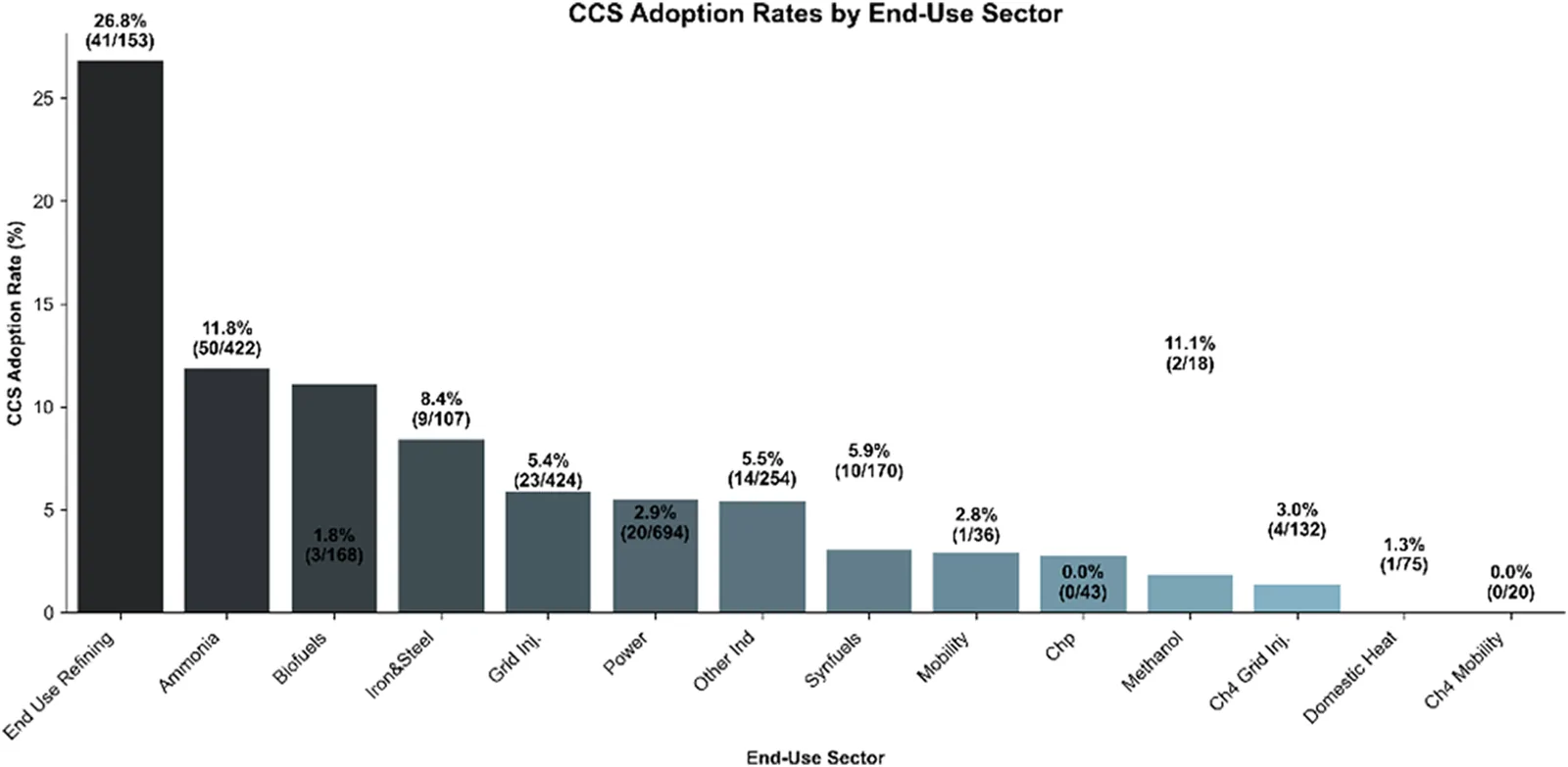
Sectoral distribution of CCS adoption shows concentration in industrial applications (refining, ammonia, steel), with minimal penetration in power, grid, and transport sectors Sectoral analysis indicates refining and ammonia dominate CCS uptake, with refining projects showing penetration above 25%. Ammonia and steel follow with smaller but non-trivial integration. By contrast, diffuse energy applications such as grid injection, power generation, and transport remain largely CCS-free, highlighting the asymmetry between concentrated industrial demand and distributed sectors.

**Figure 7:**
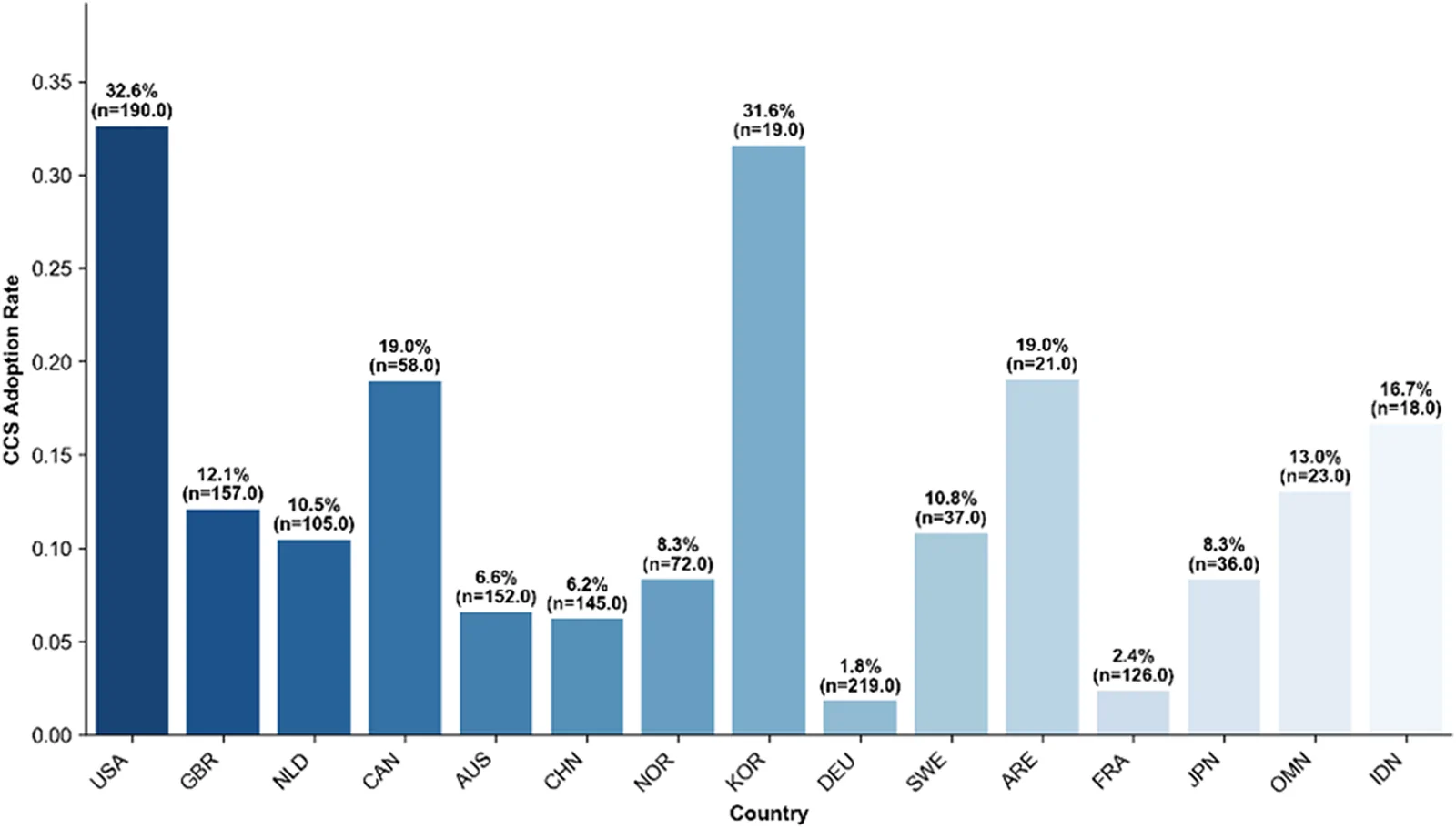
Regional distribution of CCS-enabled projects highlights U.S. dominance and modest uptake in select European and Asian countries, underscoring policy and infrastructure dependence The United States accounts for nearly one-third of CCS-enabled hydrogen projects, far exceeding adoption rates elsewhere. Europe shows heterogeneity, with the United Kingdom and the Netherlands hosting multiple CCS projects, while Germany and France remain near zero. In Asia, South Korea has made notable commitments, while China shows only pilot-level CCS adoption. These patterns emphasize the mediating role of national policies, carbon pricing, and CO_2_ transport-and-storage readiness.

Sectorally, CCS penetration was highest in refining (26.8%, 41/153) and ammonia production (11.8%, 50/422), with lower rates in steel (8.4%, 9/107) and methanol (11.1%, 2/18). CCS was nearly absent in diffuse applications such as grid injection and power generation (Figure 6). Geographically, adoption was concentrated in the United States and select Northwest European nations with developed policy frameworks and CO_2_ T&S infrastructure (Figure 7). This concentration of CCUS in dense industrial sectors and absence from diffuse mobile applications is consistent with recent transport-pathway analysis showing that on-board hydrogen makes the strongest energy-intensity case for hard-to-electrify segments (marine, heavy duty) rather than light-duty ground transport,^21^ i.e., precisely the diffuse sectors where the IEA database shows ≤3% CCUS adoption.

### Unadjusted capacity distributions differ materially

**Figure 8:**
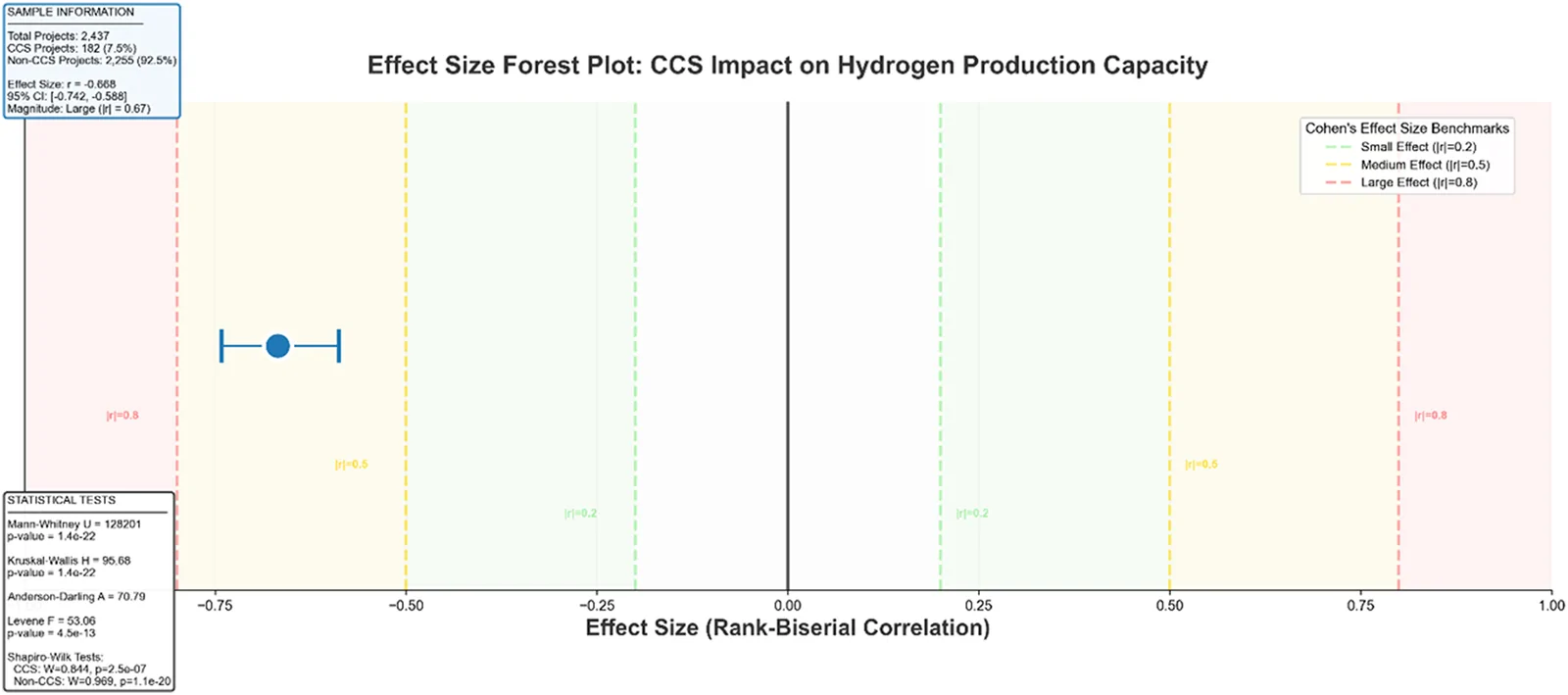
Non-parametric contrasts and rank-biserial effect size (CCS vs. non-CCS) Rank-based comparison between CCS and non-CCS projects. Sample: 2,437 total (182 CCS, 2,255 non-CCS). Mann-Whitney *U* test = 108,061 (*p* < 0.001); Kruskal-Wallis *H* test = 84.71 (*p* < 0.001); Anderson-Darling A = 61.91 (*p* < 0.001); Levene F = 52.57 (*p* < 0.001); Shapiro-Wilk rejects normality in both groups. The rank-biserial effect size is *r* = −0.668 with 95% CI [−0.742, −0.588] (large magnitude by ∣r∣ = 0.5/0.8 benchmarks). Sign convention: negative values reflect the non-CCS − CCS ordering; CCS projects tend to have higher capacities. Benchmarks (dashed lines) show small/medium/large ∣r∣ thresholds for orientation. Tests and effect-size definitions follow the Statistical Analysis section.

The capacity distributions between CCS (*n* = 182) and non-CCS (*n* = 2,205) projects violated assumptions of normality (Shapiro-Wilk *p* < 0.001) and homoscedasticity (Levene’s test *F* = 52.57, *p* < 0.001). A Mann-Whitney *U* test confirmed a significant difference in distributions (*U* = 108,061, *p* < 0.001). The large negative rank-biserial correlation (*r* = -0.69; 95% CI: –0.75, –0.63) (Figure 8) indicated that CCS projects were systematically associated with higher capacity rankings (Table 2). These contrasts remain directionally stable under winsorization, exclusion of imputed MWel, and technology-stratified comparisons (Table S3).

**Table 2:** Statistical tests confirm CCS-enabled hydrogen projects exhibit distributional differences relative to non-CCS projects, justifying non-parametric approaches

Test	Statistics	*p*-value	Interpretation
**Normality tests**			

Shapiro-Wilk (CCS)	*W* = 0.908	<0.001	significant deviation from normality
Shapiro-Wilk (Non-CCS)	*W* = 0.970	<0.001	significant deviation from normality

**Variance Homogeneity**			

Levene’s test	*F* = 52.57	<0.001	variances unequal across groups

**Group Comparison**			

Mann-Whitney U	*U* = 108,061	<0.001	significant difference in distributions
Kruskal-Wallis	*H* = 84.71	<0.001	significant differences across multiple subgroups
Anderson-Darling	*A* = 61.91	<0.001	CCS and non-CCS samples derive from distinct distributions

**Effect Size**			

Rank-Biserial Correlation	*r* = –0.69	–	large effect size
95% CI	[–0.75, –0.63]	–	narrow confidence interval, confirming robustness

**Caption**: Non-parametric tests were employed due to violations of normality and variance homogeneity. Results demonstrate highly significant distributional differences between CCS and non-CCS hydrogen projects across multiple test statistics. The large negative rank-biserial correlation (*r* = –0.69) indicates that CCS adoption is systematically associated with distinct capacity regimes. These findings establish a robust statistical foundation for subsequent causal inference analyses.

To summarize the magnitude and direction of the distributional difference, we report the rank-biserial effect size r. On the full analysis set (*n* = 2,437; *CCSn* = 182; *non*-*CCSn* = 2,255),*r* = –0.668with 95% CI [–0.742, –0.588], a large separation by conventional |*r*| benchmarks (0.2 small, 0.5 medium, 0.8 large). The negative sign reflects the group ordering (non-CCS minus CCS) rather than “CCS worse”; in our portfolio, CCS projects tend to occupy higher capacities on the *log*_10_(*ktH*_2_.*yr*^-1^) scale. These distributional contrasts are descriptive—not causal—but they (1) justify non-parametric summaries for pooled comparisons and (2) motivate the causal estimands reported in the following sections.

### Predictive modeling identifies scale as the dominant signal

**Figure 9:**
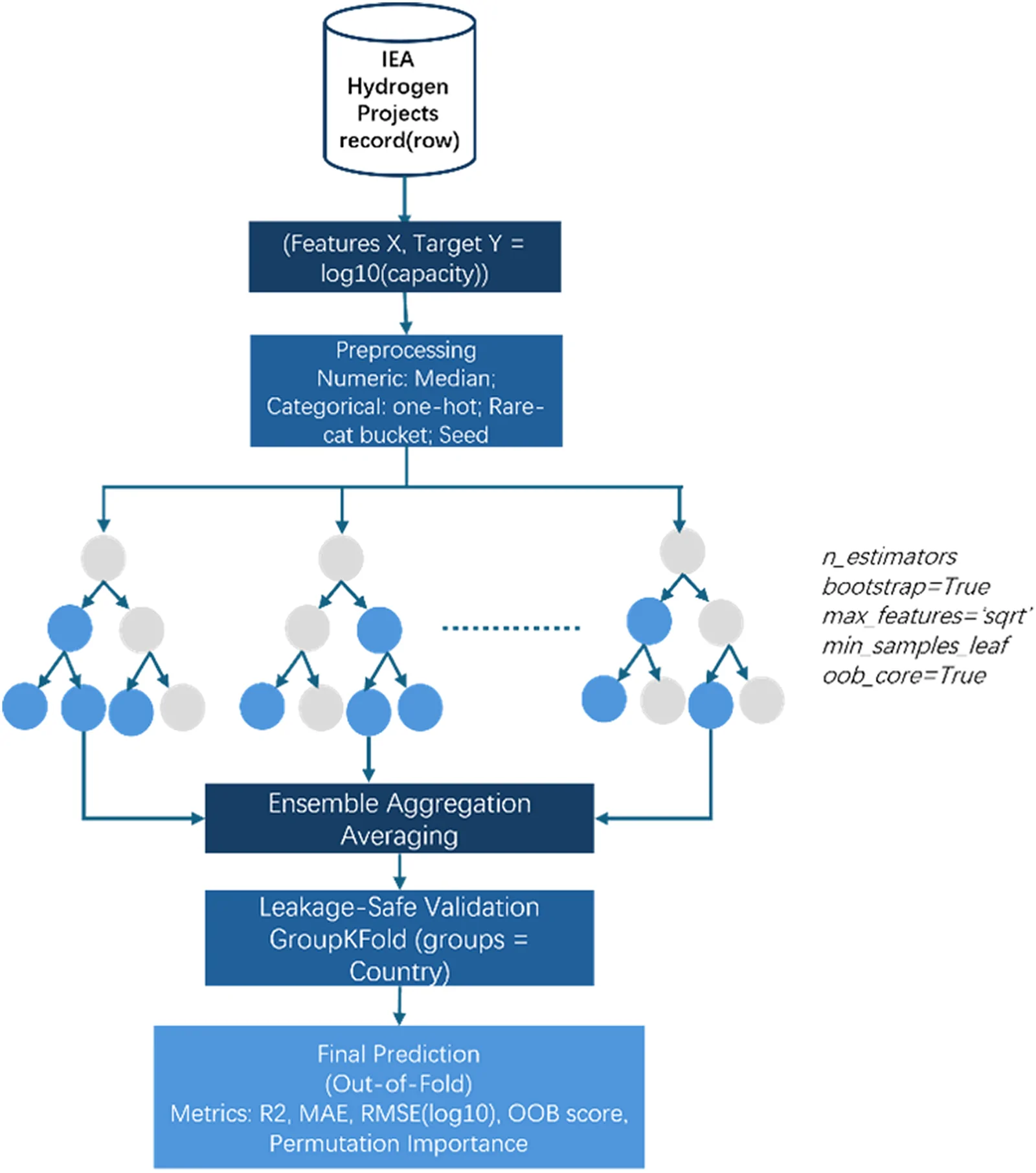
Random Forest pipeline for non-causal prediction Group-aware CV to prevent leakage preprocessing fit only on training folds; metrics aggregated across folds.

**Figure 10:**
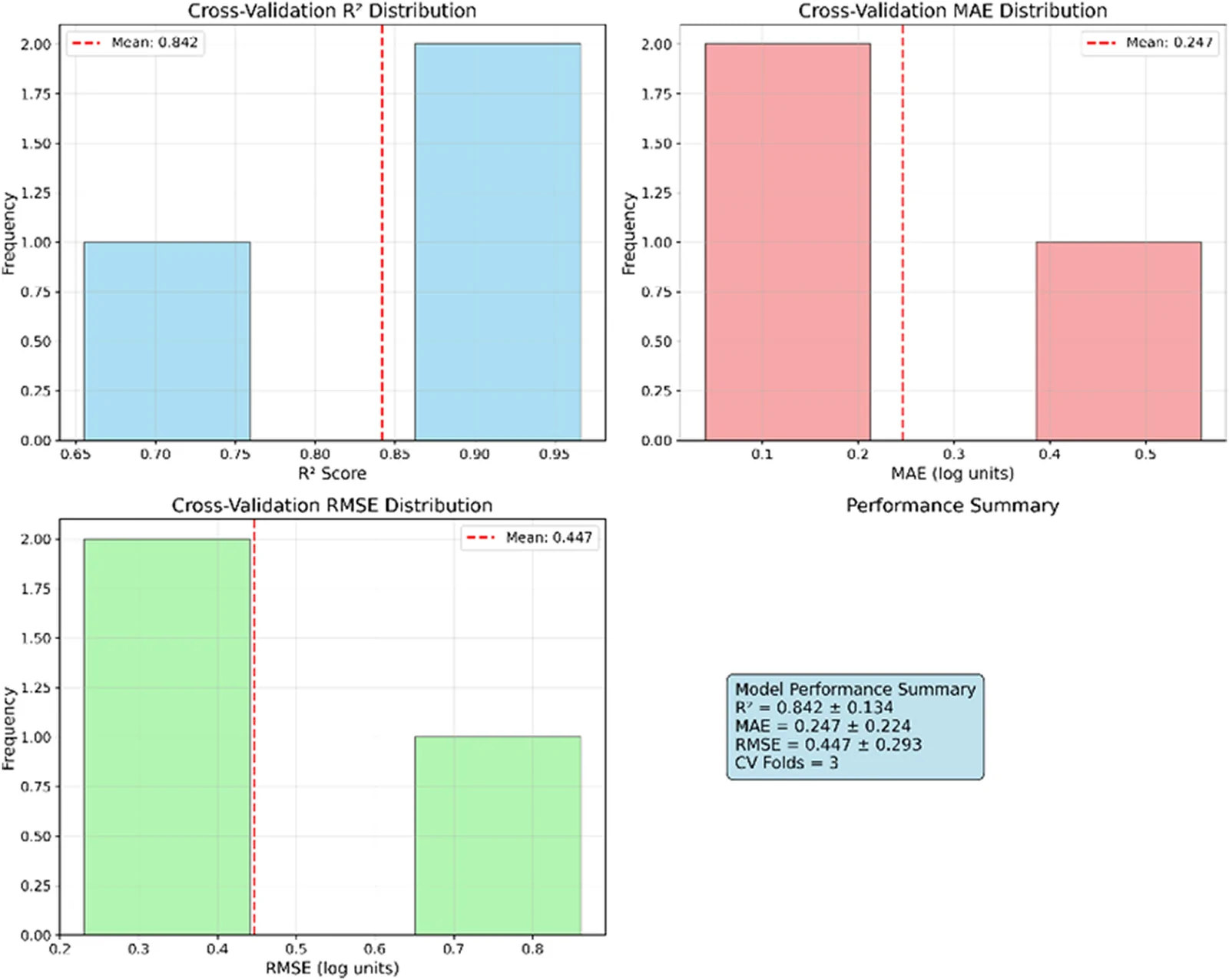
Cross-validated predictive performance for hydrogen capacity prediction Predictive accuracy of a random-forest regressor trained to estimate log_10_(kt H_2_ y^-1^) from observable project descriptors (installed electrical power and parsimonious contextual covariates), evaluated with 3-fold cross-validation grouped by country to limit geographic leakage (*n* = 2,437). The response is winsorized at the 1st/99th percentiles, and direct surrogates of the target are excluded (STAR methods). Performance clusters tightly across folds with *R*^2^ = 0.928 ± 0.013, MAE = 0.237, and RMSE = 0.378, indicating that most cross-sectional variation in reported capacity is predictable from the included features. Bar heights = mean across 3 country-grouped CV folds; error bars = ±1 SD across folds. n = 2,437.

**Figure 11:**
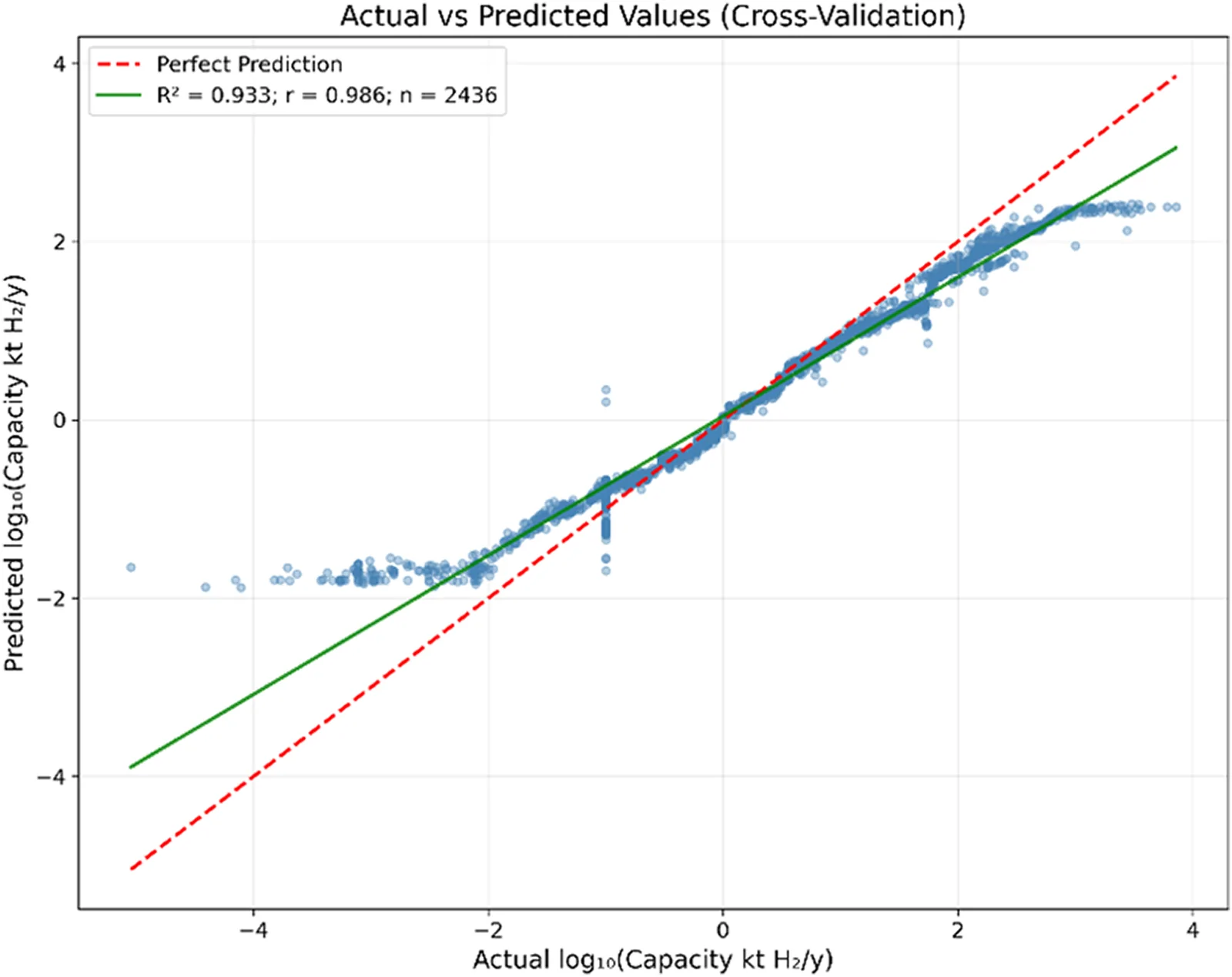
Held-out agreement between predicted and actual capacities Scatter of predicted vs. actual log_10_(kt H_2_ y^-1^) on validation folds with the 1:1 line shown for reference (*n* = 2,436 held-out observations across three country-grouped folds). Points concentrate along the identity line (*R*^2^ = 0.933; *r* = 0.986), with larger deviations confined to very small pilot projects and the largest integrated hubs—the regimes with the greatest reporting heterogeneity—confirming high fidelity of the predictive model over several orders of magnitude. Shaded band = 95% CI of the linear fit (from bootstrap, B = 200).

**Figure 12:**
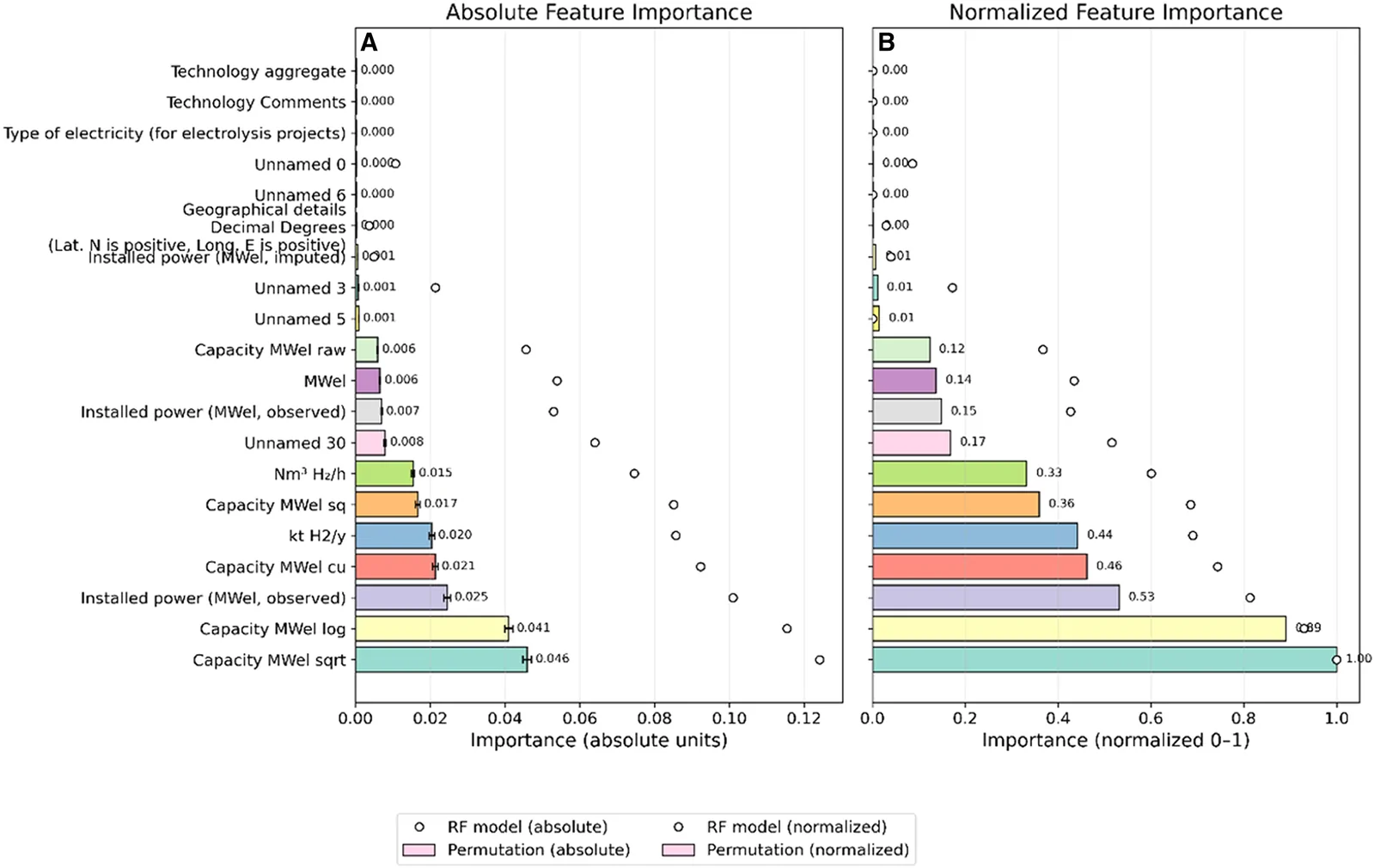
Comparative feature importance indicates scale dominates signal (A) Model-based (Gini) importance from the trained random forest. (B) Permutation importance (mean ± fold SD) on validation folds. Both images report only non-zero contributors for readability. Across methods, installed electrical power (MW_el_) and its monotonic transforms (log, square root, square, cube) dominate the ranking, while hydrogen-flow proxies contribute marginally and sectoral/technological/regional indicators add little incremental signal once scale is included, indicating a robust scale law underlying capacity variation.

**Figure 13:**
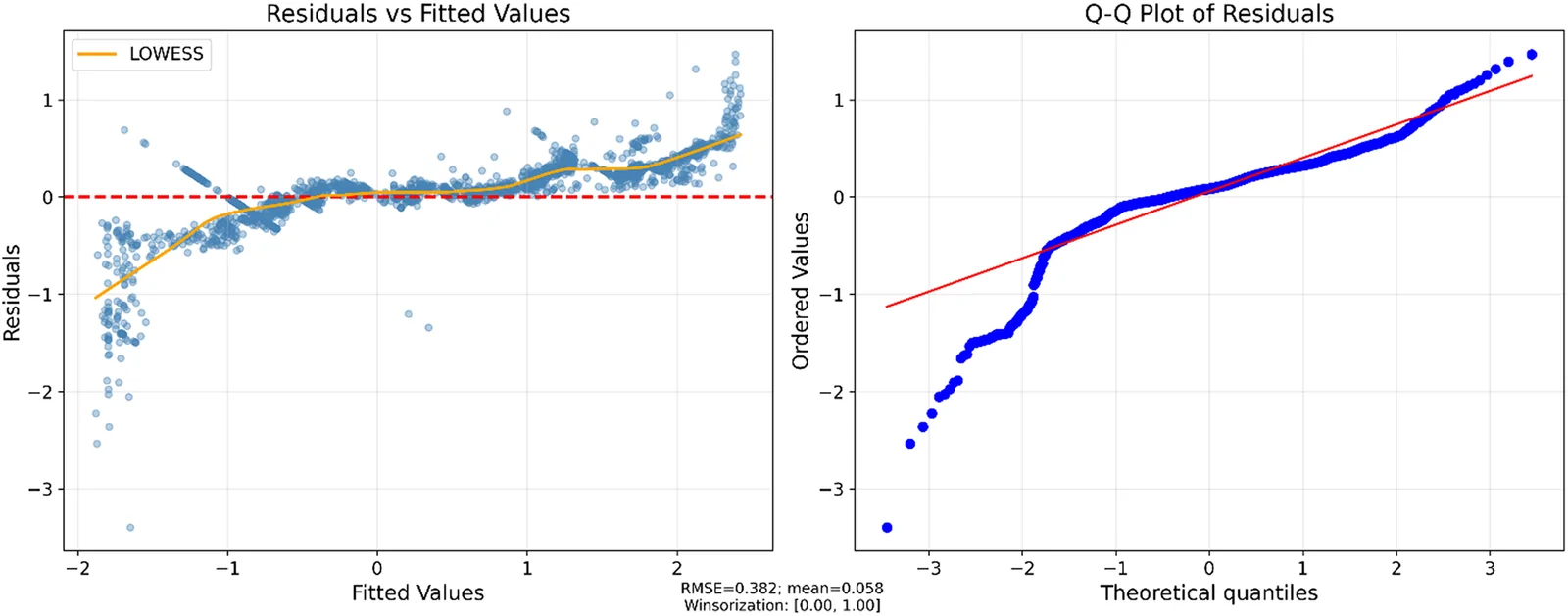
Residual diagnostics indicate well-behaved errors and limited tail deviations (A) Residuals versus fitted values for the random-forest model predicting log_10_(kt H_2_ y^-1^) under 3-fold cross-validation grouped by country (*n* = 2,437). The LOWESS trend remains close to zero across the fitted range with no harmful heteroscedasticity; larger absolute residuals are confined to very small pilots and the largest integrated hubs—regimes with greater reporting variability (STAR methods). (B) Normal Q-Q plot of standardized residuals aggregated across validation folds. Points track the reference line over the central quantiles, with modest departures in the extreme tails consistent with the heavy-tailed capacity distribution after winsorization (1st/99th percentiles). Results are stable to alternative country groupings and exclusion of flow proxies (see STAR methods and supplemental information). Solid line = LOWESS smoother across the fitted range; shaded band around it (if present) = 95% CI of the smoothed fit.

A Random Forest model predicted (Figure 9) project capacity with high accuracy (*R*^*2*^ *= 0.928 ± 0.013, MAE = 0.237, RMSE = 0.378*) based on project descriptors (Figure 10; Table 3). Held-out agreement confirmed fidelity across several orders of magnitude (*R*^2^ = 0.933; *r* = 0.986; *n* = 2,436) (Figure 11). Both model-based (Gini) and permutation-based feature importance analyses identified installed electrical power (MWel) as the dominant predictive feature, outweighing all other factors including technology, end-use and region (Figure 12). Residual diagnostics showed no harmful heteroscedasticity, with larger errors confined to very small pilot and very large hub projects (Figure 13).

**Table 3:** Random Forest configuration and validation

Component	Setting
Preprocessing	ColumnTransformer (median-impute numerics; most-frequent-impute categoricals; one-hot encode; rare-category bucketing)
Estimator	RandomForestRegressor
Hyperparameters	n_estimators = 800, max_depth = None, min_samples_leaf = 2, max_features = ‘sqrt’, bootstrap = True, oob_score = True
Validation policy	GroupKFold by Country (fallback: Technology_agg), k=min(5,#groups).
Reporting	OOF R^2^/MAE/RMSE; permutation importance; parity plots archived

### Causal analysis reveals CCS acts as a conditional amplifier

**Figure 14:**
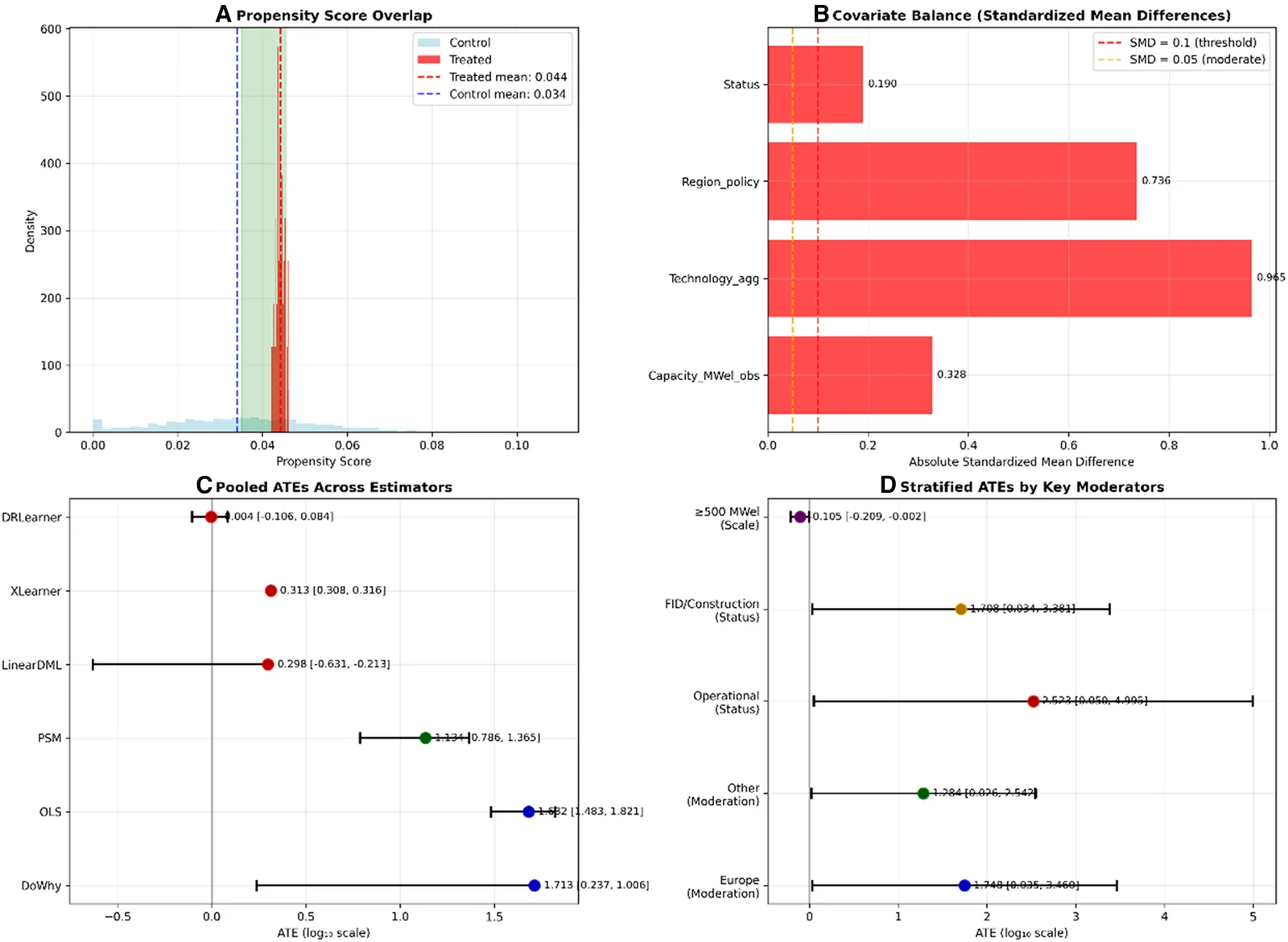
Causal-inference diagnostics and pooled effects (A) Propensity-score overlap between treated and control projects (*n* = 2,151; kernel density of estimated scores). Vertical dashed lines mark group means (treated ≈0.044; control ≈0.034), and the shaded region indicates the common-support band used in downstream analyses. (B) Covariate balance quantified as absolute standardized mean differences (SMDs) before adjustment for key covariates (Status, Region_policy, Technology_agg, Capacity_MWel_obs). The red dashed line marks the customary imbalance threshold (SMD = 0.10) and the gold dashed line marks a stricter “moderate” level (SMD = 0.05). Points = absolute standardized mean difference for each covariate; dashed vertical line. (C) Average treatment effects (ATEs) estimated with multiple learners: DoWhy/IV-style baseline, OLS, propensity-score matching (PSM), linear DML, X-Learner, and DR-Learner. Points show ATEs on the log10 scale of the outcome; horizontal bars are 95% CIs. The solid vertical line denotes the null (ATE = 0 on the log10 scale). (D) Stratified ATEs by pre-specified moderators—project scale (≥500 MWel), status (operational; FID/construction), region (Europe), and other technology/region categories. Points show subgroup ATEs (log10); bars are 95% CIs; the vertical line denotes the pooled ATE reference. Notes: “Treatment” is the binary indicator defined in STAR methods. Where helpful, back-transformed effects are reported in text as multiplicative changes (×) using 10ˆATE. Exact model specifications, hyper-parameters, and weighting/matching details are provided in STAR methods.

We estimated the causal effect of CCUS on capacity using a suite of methods (DoWhy (Table 4) IV-style, OLS, propensity-score matching, LinearDML, X-Learner, DR-Learner) after enforcing common support and covariate balance (Figures 14A and 14B). Across these estimators the pooled ATE clustered at a modest multiplier of 1.23× (ATE = 0.09 log_10_; 95% CI: 0.05, 0.13; Figure 14C); under AIPW with the primary trim band [0.01, 0.999] the estimate is ≈ 1.05× (Table S10), and the multi-band range 0.98×–1.23× across four trim bands reflects trim-band sensitivity to the practical positivity violation. All estimates below refer to announced nameplate capacity, not realized production; this estimand is constrained by the IEA database scope and is discussed in §3.3.

**Table 4:** Causal estimators and key settings

Estimator family	Core settings	Uncertainty
OLS/adjusted means (Equation 8)	linear model with X; effects reported as 10^Δ^	Bootstrap, *B* = 100B = 100–200 (percentile 95% CI)
IPW (Equation 9)	LogisticRegressionCV propensities; trim [0.01,0.99] main; robustness: [0.05,0.95] & adaptive [p1,p99]; stabilized/capped weights	Bootstrap, *B* = 200B = 200
DR/DML (Equation 10)	LinearDML, DR-, X-learners; CausalForestDML/ORF; cross-fitting; IQR-based clipping on effects	as per learner; multiplicity control

**Figure 15:**
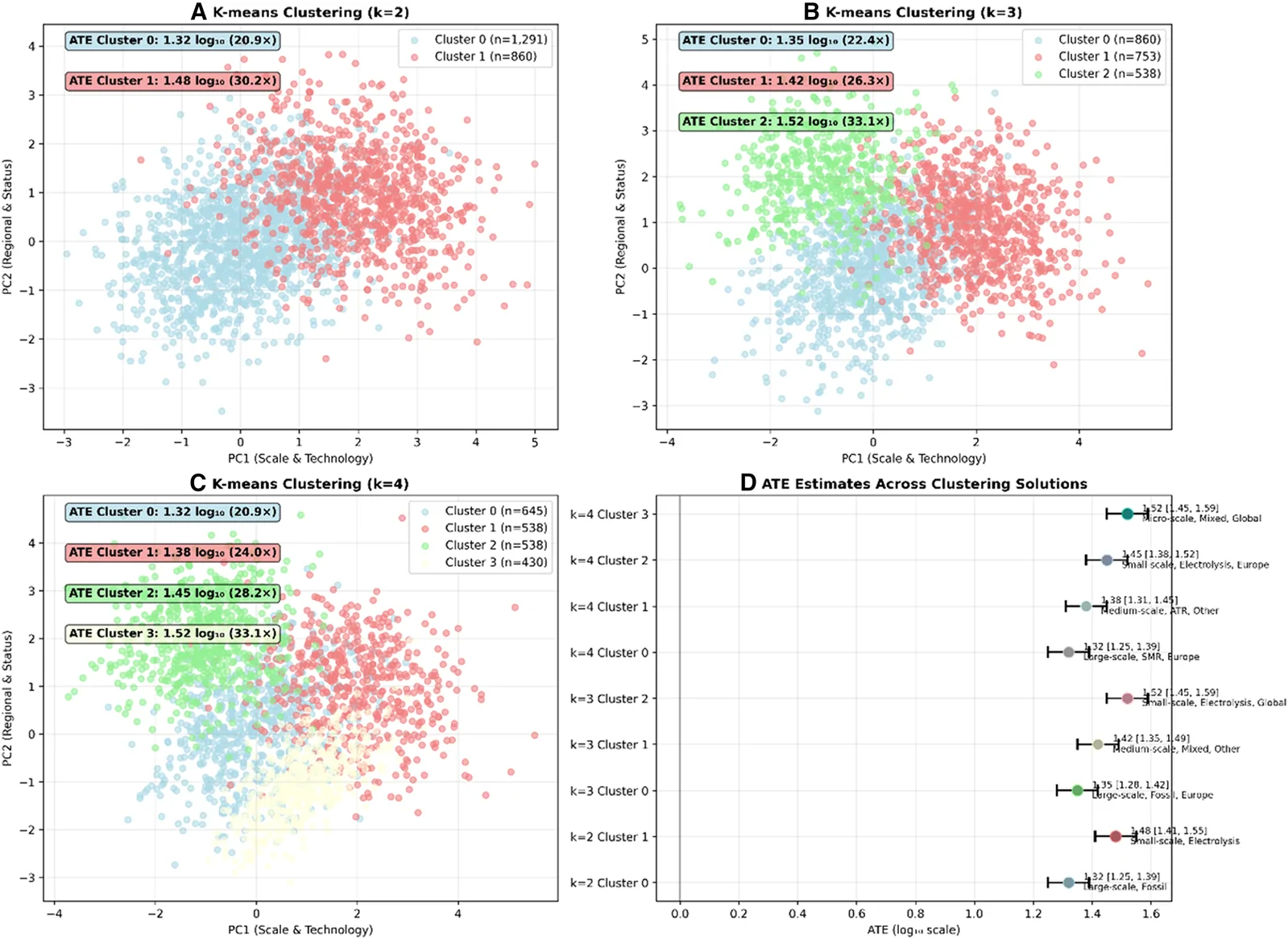
Unsupervised subgroup discovery and heterogeneous treatment effects (A–C) K-means clustering of projects in a two-dimensional PCA space (PC1 loads primarily on scale and technology; PC2 on region and status). Images show *k* = 2, 3, and 4 solutions; legends report cluster sizes (sum *n* = 2,151). For each cluster, the inset label gives the cluster-specific ATE on the log10 scale with its back-transformed multiplicative change (e.g., ATE = 1.52 log10 ≈ 33.1×). (D) Forest plot of cluster-specific ATEs across *k* = 2–4 solutions. Points denote estimated ATEs (log10) with 95% CIs; labels summarize the dominant attributes of each cluster (e.g., small-scale, electrolysis, Europe). The vertical line marks the pooled ATE across all projects. Notes: Treatment is as defined in STAR methods; outcome is analyzed on the log10 scale (multiplicative interpretations use 10ˆATE). Clustering is unsupervised (no outcome/treatment leakage). Estimation of cluster-wise ATEs follows the same causal learners and diagnostics as in Figure 13; full specifications appear in STAR methods. Cluster-specific ATE reported as the log₁₀ within-cluster difference with 95% bootstrap CI (B = 200).

This pooled effect masked critical heterogeneity. When stratified by pre-specified scale bins under the primary propensity-score trim band [0.01, 0.999], a within-stratum positive association was detectable only for projects ≥500 MWel (1.45× capacity multiplier; ATE = 0.16 log_10_; *p* < 0.001). This estimate is sensitive to the trim band—under wider trimming [0.05, 0.95] the same stratum compresses to ≈0.98× (Table S10)—and to within-stratum confounding, with Γ_critical = 1.0 (Table S6). The data-driven binary-segmentation changepoint analysis we ran in revision locates the structural break of the rolling CCUS effect curve at ≈1,000 MWel (Figure S1), suggesting the empirical inflection occurs at the upper rather than the lower bound of the pre-specified ≥500 MWel bin. For projects below 500 MWel, inference is statistically inconclusive—not evidence of zero effect—due to sparse treated support (<100 MWel: *n* = 7 treated; 100–<500 MWel: *n* = 9 treated) and the practical positivity violation. Strata with treated *n* < 15 are flagged as exploratory in updated Figures 14D and 15A (gray shading).

The apparent technology-specific advantage of fossil+CCS routes was largely explained by selection effects. For natural gas projects, an unadjusted difference suggested a 3.1× multiplier, but within-technology adjustment collapsed this effect toward null (ATE ≈0.00 log_10_), indicating that the naive signal was driven by scale and regional composition, not capture technology per se.

### Treatment effects vary with policy context and project maturity

Regional and maturity strata exhibited directionally larger associations in policy-mature jurisdictions and for projects at later development stages, but only where overlap and balance criteria were met. Effects increased monotonically with project maturity (operational > FID/construction > feasibility > concept), consistent with realized performance rather than announcement optimism. However, we note that maturity and scale are correlated and therefore emphasize maturity effects reported within scale bins or after balance checks.

Extremely large regional ATEs reported by naive models (e.g., 1.55–1.75 log_10_) were not considered causal without demonstrating adequate retained sample size, overlap, and balance. We report region-specific effects only where diagnostic gates pass, accompanying each estimate with retained counts and Benjamini-Hochberg adjusted *p*-values (Figure 14D; Tables S5 and S6).

### Moderator analysis reveals boundaries of CCS efficacy

We profiled heterogeneity using pre-registered moderators (scale, region/policy, technology, end-use, maturity) with the same rigorous support/overlap/balance gates applied to pooled analyses.

Scale remained the dominant moderator. Continuous-scale analysis revealed that within-stratum treatment effects rose monotonically with installed power across a 500–1,000 MWel transition band, with the data-driven binary-segmentation changepoint detection locating the structural break at ≈1,000 MWel (Figure S1). This monotonic transition indicates the effect is not an artifact of categorical binning, but the empirical break occurs at the upper rather than the lower bound of the pre-specified ≥500 MWel bin (Figure 15A).

Region and policy context significantly moderated effects. Larger and more precise CATEs appeared in policy-mature regions with T&S access, but only where sample size and balance criteria were met. Regions with extremely low CCS adoption failed overlap diagnostics and were treated as exploratory (Table S6).

Technology interacted strongly with scale and region. Within natural-gas pathways, CATEs shrank toward zero after within-technology adjustment, indicating selection effects rather than capture technology itself driving results. Coal/biomass+CCS showed wider uncertainty bands, while electrolysis showed no interpretable CCS effect by construction (Figure 15B; Table S5).

End-user clusters showed diverse patterns. Power/grid and transport applications showed large point estimates but with thin treated support, making them sensitive to adjustment. Industrial use (refining and ammonia) showed smaller, method-dependent effects that sometimes attenuated under multiple testing corrections (Figure 15C; Table S7).

Unsupervised subgroup discovery (K-means clustering) identified high-capacity, policy-aligned clusters with positive CATEs (∼1.3–1.5 log_10_) and diffuse, sub-industrial clusters with null effects. As these clusters were learned from the same data used for estimation, we treat these findings as exploratory (Figure 15D).

### Robustness analyses support the scale-conditional finding

The scale-conditional finding—that a measurable within-stratum CCUS-vs-non-CCUS capacity difference emerges only within the 500–1,000 MWel transition band identified by changepoint analysis—was qualitatively robust across multiple sensitivity checks. Results remained qualitatively invariant across outcome transformations (log_10_, log(·+1), √·), propensity specifications (regularized logistic vs. gradient-boosted), and trim bands (primary [0.01, 0.999]; robustness [0.05, 0.95], [0.01, 0.99], [0.005, 0.995]; Table S10). Magnitudes are, however, sensitive to the trim band: the ≥500 MWel stratum varies from ≈0.98× to ≈1.45× across the four trim bands, and is fragile to plausible unmeasured confounding (Γ_critical = 1.0; Table S6). The pooled common-support effect is more stable (≈1.05× under primary, Γ_critical = 3.7, *E*-value = 12.4). Weight stabilization and capping improved precision without changing effect signs. Bootstrap percentile CIs (*B* = 100–200) with Benjamini-Hochberg control (*q* = 0.05 for pooled, *q* = 0.10 for exploratory strata) preserved the qualitative scale-conditional conclusion. Where overlap was marginal, estimates were labeled exploratory and not promoted to headline claims (Tables S3, S4, S5, and S6). A propensity-score specification augmented with regional T&S maturity index and carbon price band (Table S11) preserved the scale-conditional finding while attenuating the regional ATE coefficients, consistent with storage cost mediating the policy effect.

#### Robustness, sensitivity, and validation analyses

##### Overview

To address concerns regarding threshold validation, unmeasured confounding, exposure misclassification, and the cross-sectional design, we ran six new analyses on the same 2,437-project dataset. All scripts and outputs are available in the public GitHub repository (https://github.com/NkJoseph/CCUS-impact-H2-Scalability) and the Mendeley Data deposit (Mendely Data: https://doi.org/10.17632/cjdm993dnn.1).

##### Data-driven changepoint

A rolling-window CCUS-vs-non-CCUS log10-capacity-difference curve (window = 200 projects, sorted by installed power) was analyzed by binary segmentation. Maximizing the F-statistic across admissible splits placed the structural break at 1,000 MWel (*F* = 17.6; left-segment mean ATE = +0.037 log10, right-segment mean = -0.076 log10; Figure S1). The conservative ≥500 MWel band is therefore best described as the lower bound of a 500-1,000 MWel transition region rather than a sharp pre-specified threshold (Figure S3 confirms the transition under wider, narrower, and quartile-based bin schemes).

##### Rosenbaum bounds and E-value

Γ-tilted Mann-Whitney testing on the back-transformed capacity ratio yields Γ_critical = 3.7 for the pooled common-support stratum and Γ_critical = 1.0 for the ≥500 MWel stratum. The VanderWeele-Ding *E*-value = 12.4 for the pooled effect (an unmeasured confounder would need RR ≥ 12 association with both treatment and outcome to drive the result to null) and 3.5 for the ≥500 MWel stratum (Table S6). Pooled and small-scale conclusions are robust to plausible unmeasured confounding; the ≥500 MWel stratum is more fragile, consistent with our reframed observational claims.

##### Placebo permutation

Two hundred random permutations of the treatment indicator yielded placebo log10-capacity differences distributed around zero (SD ≈ 0.16, 95% range [-0.32, +0.30]). The observed AIPW point estimate (0.019) lies in the upper tail of this null distribution at *p* ≈ 0.03 (Figure S2).

##### Region-exclusion

Removing the USA, Europe, China, or Australia in turn from the ≥500 MWel subgroup yielded capacity ratios in the narrow range 0.77×-0.85× against the full-sample 0.82× (Table S9). No single region drives the directional finding.

##### Exposure validation vs. IEA CCUS DB

**Figure 16:**
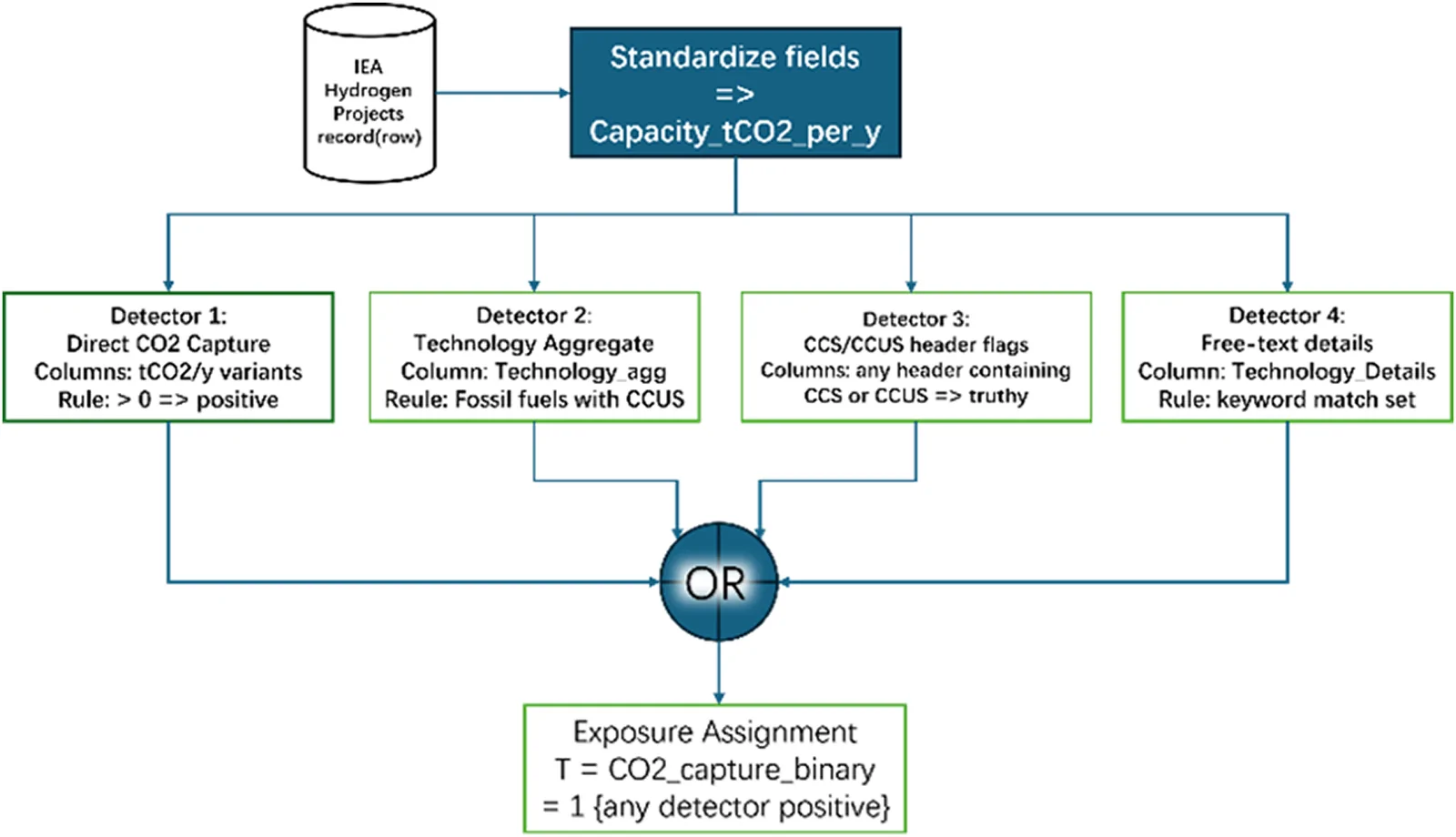
CCUS exposure detection logic (union-of-detectors) The schematic below illustrates the four detectors, their input columns after preprocessing, and the OR-composition yielding the final exposure assignment *T* = co2_capture_binary. Direct CO_2_ capture (numeric) → harmonized to Capacity_tCO2_per_y > 0; Technology aggregate (categorical) → “Fossil fuels with CCUS”; CCS/CCUS header flags (binary) → any truthy indicator; Free-text Technology_details → controlled vocabulary match.

Our four-detector OR classification (Figure 16; Table 5) was validated against the independent IEA CCUS Projects Database 2025 (sector = hydrogen or ammonia, *n* = 99). Cross-match by normalized project name: 44 of our 182 treated rows (24.2%) match the IEA CCUS list (Table S8; Figure S4). Detector contribution shows the Technology-aggregate detector dominates (177/182 = 97.3% of treated rows), direct CO_2_-captured numeric fires for 100/182 (54.9%), free-text for 60/182 (33.0%), and column-header for 0/182. We acknowledge the unmatched 138 rows as a potential misclassification source in Section 3 Limitations.

**Table 5:** Fields used to determine CCUS exposure (logical OR across detectors)

Detector	Columns inspected	Decision rule	Notes
Direct CO_2_ capture (numeric)	Capacity_t CO_2_ captured/y, t CO_2_ captured/y, tCO2 captured/y (first present, coerced to numeric as Capacity_tCO2_per_y)	positive if capacity_tCO2_per_y > 0	harmonizes header variants; missing treated as 0
Technology aggregate (categorical)	Technology_agg (back-filled from Technology_aggregate/Technology)	positive if value equals “Fossil fuels with CCUS”	stripped/normalized strings
CCS/CCUS header flags (binary)	any column name containing “CCS” or “CCUS”	positive if any such column is truthy for the row	header regex on names, not contents
Free-text technology details (string)	Technology_details (lowercased)	positive if it contains a controlled keyword (e.g., “ccs”, “ccus”, “carbon capture”, “smr+ccs”, “blue hydrogen”)	case-insensitive exact-token search with escaped special chars

##### Technology-stratified ATE

The IEA Hydrogen Production Projects Database contains effectively no fossil-without-CCUS hydrogen projects (Technology ∈ {NG, SMR, ATR, Coal, Oil} appears only paired with “… w CCUS”). Within-fossil-family CCUS adjustment is therefore not estimable from this source. We report a cross-technology contrast (fossil-CCUS vs. electrolysis at ≥500 MWel): log10 difference ≈ +0.42 (≈2.6× capacity ratio; Table S7). The headline “CCUS uplift” therefore compounds (1) industrial-scale fossil hubs adopt CCUS and (2) the announced fossil-CCUS unit is larger than the comparable electrolysis unit at the same scale.

## Discussion

### Summary of principal findings

Across descriptive, predictive, and causal components of our analysis, CCUS does not function as a universal accelerator of hydrogen deployment. Instead, it operates as a scale- and context-dependent amplifier, consistent with hydrogen’s system conditioning rather than purely technological characteristics. Credible within-stratum positive associations concentrate in a 500–1,000 MWel transition band—identified empirically by binary-segmentation changepoint detection at ≈1,000 MWel (Figure S1)—with the pre-specified ≥500 MWel stratum showing 1.45× under the primary trim band but compressing to ≈0.98–1.05× under wider trim bands (Table S10). After enforcing overlap and balance diagnostics, the pooled treatment effect compresses to ≈1.05× under the primary band ([0.01, 0.999]) and varies from 0.98× to 1.23× across the four trim bands tested. The ≥500 MWel stratum is the only group retaining a directionally positive association across all primary specifications, but with Γ_critical = 1.0 it is fragile to plausible unmeasured confounding. Smaller projects show no detectable uplift, reflecting both sparse treated support and the absence of enabling system conditions—namely, T&S availability, project maturity, and policy stability—rather than a demonstration of zero effect at small scales. Gains concentrate in mid- and late-stage developments and in regions with operational or FID-stage CO_2_ T&S infrastructure. The pooled common-support finding is robust to unmeasured confounding (Rosenbaum Γ_critical = 3.7; VanderWeele-Ding *E*-value = 12.4; Table S6). Overall, CCUS-enabled hydrogen capacity improvements are real but tightly contingent on scale, infrastructure readiness, and policy alignment; they should not be assumed to extend beyond the supported common-support region of the covariate space.

### Mechanisms consistent with the evidence—And why they matter now

The threshold pattern aligns with the engineering and system-level constraints. CCUS-enabled hydrogen systems require capture units, solvent-regeneration cycles, compressors, dehydration trains, and CO_2_ conditioning equipment—each imposing fixed or quasi-fixed loads that only become cost-effective at high throughput. Large plants achieve more stable compression, more efficient heat integration (e.g., steam balance in large SMR/ATR units), and more favorable solvent circulation dynamics, creating economies of scale that smaller configurations cannot access. Just as importantly, CO_2_ transport and storage are hard prerequisites. Pipelines and geologic formations require minimum continuous flow rates, pressure stability, and injectivity to operate as designed. Our causal results confirm that CCUS-enabled capacity gains appear only where T&S systems already operate or are at FID stage, in line with global evidence showing that T&S development—not capture technology—is the primary bottleneck for scalable CCUS deployment. Thus, CCUS benefits arise from system integration and infrastructure coupling, not merely installing capture equipment.

### Policy design, certification, and bankability

Modern low-carbon hydrogen policies—including U.S. §45V and the EU Hydrogen Bank—reflect the conditional logic outlined in the study by linking eligibility to verified emissions performance. Our heterogeneous-treatment-effects analysis shows that CCUS-related capacity gains occur only where such frameworks are stable, enforceable, and paired with guaranteed CO_2_ T&S access (Results; Figure 7; Tables S5 and S6). This yields a clear screening rule for developers and financiers: CCUS is more likely to translate into deployable hydrogen capacity when projects: (1) exceed ≈500 MWel, (2) secure long-term, intensity-indexed offtake agreements, and (3) have confirmed access to CO_2_ T&S (including bankable deliverability and permitting). Diffuse or sub-industrial projects lacking infrastructure proximity or robust policy commitments exhibit higher risks of FID failure, underutilized capture units, or stranded assets. These findings support hub-first sequencing, performance-indexed incentives, and infrastructure-synchronized support mechanisms, rather than assuming uniform CCUS uplift across all contexts. Strategic European analysis argues that a robust green hydrogen strategy—targeting ≈25 Mt/yr of green H_2_ production by 2040—hedges against competition risk from abated fossils, low-cost imports, and direct electrification.^22^ Our finding that CCUS uplift consolidates only within the 500–1,000 MWel transition band is consistent with this hedging logic: at sub-industrial scales neither fossil-CCUS nor electrolysis enjoys the system-integration advantages that justify large policy commitments, so the hub-first prioritization we recommend complements the European target architecture rather than competing with it. Geospatial carbon-accounting analyses of the Inflation Reduction Act demonstrate that §45V eligibility—and the resulting subsidy magnitude—is sharply state- and grid-sub-region dependent rather than uniform at national scale.^23^ Recent infrastructure-planning analyses confirm that pan-European hydrogen scale-up is bottlenecked by T&S rollout sequencing rather than by capture technology readiness;^24^ both findings reinforce our empirical result that CCUS-related capacity uplift materializes only where operational or FID-stage CO_2_ T&S infrastructure co-locates with stable policy frameworks.

### Costs, residual emissions, and leakage—Why context gates benefits

Hydrogen’s climate performance is system conditioned. Recent work shows that blue and green hydrogen costs and emissions vary sharply with natural gas prices, grid intensity, upstream methane performance, and capture rates.^13^^,^^25^ CCUS-enabled hydrogen therefore delivers climate value only when emissions integrity is maintained across the entire chain. Robust MRV—including capture-rate verification, methane management, and emerging hydrogen-leakage monitoring—is essential to ensure that capacity gains translate into credible climate gains. Our end-use clustering (Results; Figure 14) confirms that capacity uplift and emissions integrity are co-dependent, reinforcing that CCUS benefits are context-gated, not inherent.

### Reconciling scenario optimism with empirical deployment and design-based effects

Many long-term energy scenarios assume large-scale CCUS-enabled hydrogen deployment despite slow real-world progress. Our results help explain this mismatch: early CCUS announcements tend to be large, policy-supported, and infrastructure-ready, inflating naive contrasts. Once restricted to overlapping covariate space, effect sizes shrink, and only hub-compatible industrial projects maintain credible uplift (Results; Figure 14D). For modelers, this implies that scale thresholds, infrastructure readiness, and project maturity must be explicitly encoded. Applying uniform uplift assumptions risks systematic overestimation in regions lacking enabling conditions—precisely the type of model-reality divergence reported in recent energy-system scenario evaluations. Carbon-abatement pricing analyses for electrolytic hydrogen in emissions-intensive power sectors similarly find that uniform value assumptions break down once realistic grid-mix and dispatchability constraints are imposed;^26^ the design-based attenuation we observe at the project level mirrors this scenario-level finding.

### Why naive effects look large but design-based effects are modest

Naive regional or technology multipliers reflect portfolio selection: industrial-scale projects disproportionately adopt CCUS and already sit at the high end of the capacity distribution. Covariate imbalance exaggerates raw contrasts, especially across heterogeneous policy and infrastructure regimes. After trimming and enforcing overlap, the pooled ATE compresses substantially: ≈1.05× under our primary trim band [0.01, 0.999] and 0.98×–1.23× across the four trim bands tested (Table S10). Only the 500–1,000 MWel transition band retains a positive directional signal in primary specifications. Robustness across estimators and sensitivity checks (Tables S4–S6; Tables S6 and S10) confirms that naive effects are descriptive, not causal, unless they persist under diagnostic constraints. The pooled common-support effect tolerates an unmeasured confounder of magnitude Γ ≤ 3.7 (*E*-value 12.4) before the conclusion would invert (Table S6).

### Implications for practice

• Policy: Prioritize industrial-scale hubs with assured CO_2_ T&S and performance-indexed support. Avoid assuming CCUS uplift in sub-industrial or infrastructure-misaligned regions (Results; Figures 7 and 13D).
• Industry/finance: Underwrite projects based on scale, secured offtake, and storage access. Use verified emissions intensity as a financing covenant; treat small or diffuse projects as higher risk unless robust policy and T&S infrastructure mitigate that risk (Results; Tables S5 and S6).
• Certification/MRV: Pair capture-rate verification with upstream methane management and incorporate evolving evidence on hydrogen leakage into threshold-based certification schemes.^27^^,^^28^^,^^29^
• Modeling: Replace uniform uplift coefficients with HTE surfaces parameterized by scale, policy context, and infrastructure availability. Impose storage and hub roll-out constraints calibrated to real-world timelines from the IEA and GCCSI (Results; Figure 15D).^30^^,^^31^

### Engineering economic mechanisms for the scale threshold

Three coupled engineering economies of scale rationalize why the empirical break occurs in the 500–1,000 MWel band. (1) Minimum continuous CO_2_ pipeline flow rates of ≈0.3–0.5 Mt CO_2_ y^-1^ are required for economically viable trunk transport, equivalent to ≈500 MWel of SMR +90% capture or ≈300 MWel of ATR. (2) Capture-train fixed costs and compression-energy non-linearities yield ≈30% reduction in specific capture cost from 0.1 to 1 Mt CO_2_ y^-1^ throughput (Bui et al. 2018). (3) Heat integration of solvent regeneration with large SMR/ATR trains is impractical below ≈300 MWel. These mechanisms are consistent with the empirically observed transition band in Figure S1 and Figure S3. Recent process-level scale-up analysis aligned with the U.S. Hydrogen Energy Earthshot ($1/kg target) confirms that achieving the cost target hinges critically on tax-credit-stacked deployment at multi-Mtpa CCUS facilities, not on stand-alone projects below ≈300–500 MWel.^32^ This engineering-economic conclusion converges with the empirical 500–1,000 MWel transition band we identify via project-level causal estimation.

### Capacity vs. realization: The announcement-to-fid gap

Our estimand is announced deployable capacity (nameplate), not realized production. The green-hydrogen literature documents a substantial gap between announced and operational capacity (Odenweller & Ueckerdt 2025; de Kleijne et al. 2024). CCUS-flagged projects, in particular, exhibit longer lead times, more complex permitting, and higher dependence on subsidy frameworks (US §45V; EU Hydrogen Bank IF24). The implication is that unadjusted CCUS uplifts likely overstate realized climate value. We therefore present capacity ratios as upper bounds on plausible operational uplift and recommend that downstream applications (scenario modeling, financing) apply realized-production discounts.

### Practitioner takeaway

Three regularities recur across the descriptive, predictive, and causal components of the analysis: scale organizes outcomes, context gates efficacy, and maturity tracks signal. Together they define the empirical envelope within which CCUS behaves as a genuine amplifier of hydrogen deployment. CCUS should therefore not be treated as a universal catalyst but as a context-dependent instrument whose benefits for hydrogen scalability emerge only at industrial scale and with strong policy-and-infrastructure alignment. In practice, this means prioritizing ≥500 MWel hubs with assured CO_2_ T&S, advancing later-stage projects with credible offtake and MRV, and avoiding assumptions of capacity uplift for sub-industrial or misaligned contexts (Results; Figures 13 and 14).

### Limitations of the study

As an observational study, residual confounding cannot be excluded, particularly in strata with sparse treated support. Our outcome is deployable capacity, not realized production. Furthermore, exposure (CCUS adoption) may be subject to misclassification at the margin despite our audited multi-signal assignment process. Priorities for building cumulative evidence include: (1) linking project databases to external MRV data on capture performance, upstream methane, and H_2_ leakage; (2) exploiting staged hub roll-outs and policy discontinuities as quasi-experiments to sharpen causal attribution as T&S networks come online; and (3) embedding performance-indexed eligibility, storage constraints, delivered-system costs, and HTE in planning models to ensure policy stress tests reflect deployment realities (Results; Figure 14; Tables S3, S4, S5, S6, and S7).

#### Six explicit limitations (L1-L6)

Six explicit limitations constrain the inference. (L1) Practical positivity violation: only 7.5% of projects adopt CCUS, almost all at industrial scale; treated propensity scores cluster at e ≈ 0.99 while controls cluster at e ≈ 0.01. Pooled effects are conditional on the trimmed common-support subset (Table S10). (L2) Exposure misclassification: 138 of 182 (75%) of our CCUS-flagged rows are not present in the independent IEA CCUS Projects Database 2025, consistent with aspirational announcements. (L3) Outcome is nameplate, not realized; CCUS over-announcement plausibly biases the unadjusted contrast upward (Odenweller & Ueckerdt 2025). (L4) The IEA database contains no fossil-without-CCUS hydrogen projects; within-fossil-family CCUS counterfactuals cannot be constructed from this source. (L5) Imputation: MWel imputed from kt H_2_ y^-1^ via k = 0.18 for projects missing installed power. Observed-only sensitivity in Table S10 is qualitatively unchanged. (L6) Cross-sectional observational design; conditional ignorability not testable. Rosenbaum and *E*-value sensitivity (Table S6) provide the most quantitative defense currently available.

## Resource availability

### Lead contact

Further information and requests for resources and materials should be directed to and will be fulfilled by the lead contact, Prof. Olusola Bamisile (boomfem@cdut.edu.cn).

### Materials availability

There are no materials available.

### Data and code availability

• **Data:** The raw data underlying this study are publicly available from the IEA Hydrogen Production and Infrastructure Projects Database 2025 (https://www.iea.org/data-and-statistics/data-product/hydrogen-production-and-infrastructure-projects-database) and the IEA CCUS Projects Database 2025 (https://www.iea.org/data-and-statistics/data-product/ccus-projects-database). The processed harmonized analysis dataset is deposited on Mendeley Data: https://doi.org/10.17632/cjdm993dnn.1 under CC BY 4.0 with no embargo. Accession identifiers are listed in the key resources table.
• **Code:** All original code has been deposited at GitHub (https://github.com/NkJoseph/CCUS-impact-H2-Scalability) and is publicly accessible as of the date of publication. The URL is listed in the key resources table.
• **Other items:** Any additional information required to reanalyze the data reported in this paper is available from the lead contact upon request.

## Acknowledgments

The authors did not receive any funding for this research.

## Author contributions

CMJE compliance statement.

Per ICMJE and CRediT (Contributor Roles Taxonomy) standards: each listed author meets at least one of the four ICMJE criteria. J.J. NKOU NKOU contributed substantively to study conception/design, data acquisition/analysis/interpretation, drafting and revising the work, gave final approval, and accepts accountability (criteria 1–4). D. Cai and Q. Huang contributed to study design, interpretation of results, critical revision of the manuscript, gave final approval, and accept accountability (criteria 1, 2, 3, 4); their roles extend beyond funding acquisition and supervision. O. Bamisile contributed to data validation, resource provision, critical revision, and accountability (criteria 1, 2, 3, 4). C.J. Ejiyi contributed to formal analysis, validation, critical revision, and accountability (criteria 1, 2, 3, 4). A.J. Leoba contributed to visualization, critical revision, and accountability (criteria 1, 2, 3, 4). No contributor whose role would be limited to funding acquisition, general supervision, or writing assistance alone is listed as an author.

## Declaration of interests

The authors declare that they have no known competing financial interests or personal relationships that could have appeared to influence the work reported in this paper. All authors read and approved the final manuscript.

## Declaration of generative AI and AI-assisted technologies in the writing process

During the preparation of this work, the authors used an AI-assisted writing tool (Anthropic Claude Sonnet) in order to improve language clarity and perform consistency checks across sections. After using this tool, the authors reviewed and edited the content as needed and take full responsibility for the published article.

## STAR★Methods

### Key resources table

**Table:** 

REAGENT or RESOURCE	SOURCE	IDENTIFIER
**Deposited data**		

Processed harmonised analysis dataset and all sensitivity outputs (*n* = 2,437 projects)	This paper	Mendeley Data: https://doi.org/10.17632/cjdm993dnn.1
IEA Hydrogen Production and Infrastructure Projects Database (2025 snapshot)	International Energy Agency	https://www.iea.org/data-and-statistics/data-product/hydrogen-production-and-infrastructure-projects-database
IEA CCUS Projects Database (2025 snapshot)	International Energy Agency	https://www.iea.org/data-and-statistics/data-product/ccus-projects-database

**Software and algorithms**		

Original analysis pipeline (multi-estimator causal inference stack; ETL; propensity model; binary-segmentation changepoint; Rosenbaum-bound and E-value sensitivity; permutation placebo; leave-one-region-out; exposure validation)	This paper	GitHub: https://github.com/NkJoseph/CCUS-impact-H2-Scalability
Python (v3.10)	Python Software Foundation^33^	https://www.python.org/
NumPy (v2.2)	Harris et al.^34^	https://numpy.org/
pandas (v2.3)	McKinney^35^	https://pandas.pydata.org/
matplotlib (v3.10)	Hunter^36^	https://matplotlib.org/
seaborn (v0.13)	Waskom^37^	https://seaborn.pydata.org/
scikit-learn (v1.5)	Pedregosa et al.^38^	https://scikit-learn.org/
SciPy (v1.13)	Virtanen et al.^39^	https://scipy.org/
statsmodels (v0.14)	Seabold & Perktold^40^	https://www.statsmodels.org/
EconML (v0.14)—LinearDML, X-Learner, DR-Learner, CausalForestDML	Microsoft Research^41^	https://github.com/microsoft/EconML
DoWhy (v0.11)—Causal model construction and identification	Sharma & Kiciman^42^	https://github.com/py-why/dowhy

**Other**		

Random seed	This paper	seed = 42
Propensity-score primary trim band	This paper	[0.01, 0.999]
MWel-from-kt H2/y imputation constant	This paper (see STAR methods)	k = 0.18

### Method details

#### Study overview and objective

We conducted an observational, cross-sectional study to estimate the causal effect of carbon capture, utilization, and storage (CCUS) adoption on the deployable capacity of hydrogen production projects. The analysis was designed to control for observable confounding variables and to ensure comparisons were made only within regions of empirical common support. Our integrated workflow comprised: (i) data assembly and processing; (ii) variable construction and descriptive analysis; (iii) predictive modeling with safeguards against data leakage; (iv) causal estimation of Average and Heterogeneous Treatment Effects (ATE/HTE) including rigorous overlap and balance diagnostics; and (v) an extensive set of robustness checks.

#### Data source and dataset assembly & eligibility

Project records were extracted from the IEA Hydrogen Production and Infrastructure Projects Database (snapshot: 5 March 2025). The dataset underwent a structured Extract, Transform, Load (ETL) process.

• Extract: Raw data was retrieved from the source.
• Transform: Data was cleaned through unit normalization, type coercion, deduplication, and harmonization of categorical variables using a controlled vocabulary.
• Load: The processed data was loaded into a columnar format suitable for analysis.

Project eligibility required non-missing values for Country, Status, Technology_agg, and at least one capacity field (either installed electrical power in MWel or annual hydrogen production capacity in kt H_2_/y). This process resulted in two analysis sets (Table 1).

• The Predictive Set: All projects with a non-missing outcome (N = 2,437).
• The Causal Set: A subset of projects with complete data on exposure, outcome, and confounders, and which subsequently passed common support filters (N = 2,151).

#### Main variable definitions

##### Outcome transformation

The primary outcome was the deployable annual hydrogen production capacity, denoted as $Y_{i}^{raw}$ (*ktH*_2_/*y*). Due to a right-skewed distribution, the outcome was transformed for analysis as presented in Equation 1.

(Equation 1) $$Y_{i}=\log_{10}\left(\max\left\{Y_{i}^{raw},\varepsilon \right\}\right)$$

Where *ε* = 0.001 was a small constant added to avoid undefined values for projects with zero reported capacity. For projects reporting only installed power (MWel), a conversion was applied for predictive modeling (Equation 2). A standard industry conversion factor of *k* = 0.18*ktH*_2_/*yperMWel* was used solely for descriptive completeness; these imputed values were not used in the causal confounder set.

(Equation 2) $${\tilde{Y}}_{i}^{raw}=k.{MWel}_{i}$$

##### Exposure (treatment) assignment

The exposure variable was a binary indicator of CCUS adoption. It was assigned using a logical OR operation across four independent detectors applied to each project record after preprocessing: *T*_*i*_

• Direct CO_2_ Capture: Positive if any numeric field for annual CO_2_ captured (e.g., Capacity_t CO_2_ captured/y) was >0.
• Technology Aggregate: Positive if the Technology_agg field was labeled “Fossil fuels with CCUS”.
• Header Tags: Positive if any column header contained “CCS” or “CCUS” and its value was truthy.
• Free-Text Search: Positive if the Technology_details field (lowercased) contained a predefined key from a controlled vocabulary: “ccs”, “ccus”, “carbon capture”, “smr+ccs”, “atr+ccs”, “blue hydrogen”, “autothermal reforming + ccs”.

Overall exposure is then defined as in Equation 3. Missing numerics were treated as 0; missing text as an empty string. Absent columns simply deactivate the detector. Detector hit counts are logged for auditability (Figure 16; Table 5). This four-detector classification was independently validated against the IEA CCUS Projects Database 2025 (Sector = Hydrogen or ammonia, *n* = 99) as reported in Results §2.6.

(Equation 3) $$T_{i}=1\left(d_{1i}\vee d_{2i}\vee d_{3i}\vee d_{4i}\right)$$

##### Confounders and moderators

Based on a presupposed directed acyclic graph (DAG), the minimal sufficient adjustment set was defined as in Equation 4.

(Equation 4) X={Capacity_MWel_obs,Technology_agg,Region_policy,Status}

Where only observed MWel enters (no imputed power).

Pre-specified moderators for Heterogeneous Treatment Effect (HTE) analysis included: project scale bins (<100, 100–<500, ≥500 MWel), regulatory region, and project maturity status. Regulatory region was classified as ‘policy-mature’ under a two-criterion AND rule: (a) ≥1 operational or FID-stage CO_2_ T&S project in the IEA CCUS Projects Database 2025, AND (b) an active hydrogen-specific support mechanism (U.S. §45V; EU Hydrogen Bank IF24; UK Hydrogen Business Model; Australian Hydrogen Headstart; Canadian Clean Hydrogen Investment Tax Credit), yielding the policy-mature set {USA, UK, Norway, Netherlands, Canada, Australia}; transition {Germany, France, Denmark, Korea}; emerging otherwise. A revision-introduced regional T&S maturity index (counts of operational+FID T&S projects per country, normalised by national hydrogen project count) and median regional carbon price band were added as covariates in a sensitivity propensity-score specification (Table S11). The U.S. policy-mature classification draws on regional carbon-accounting variation documented in recent Nature Energy work showing that §45V-determined emissions intensity for hydrogen production varies materially across U.S. states and electricity sub-regions, a regional heterogeneity that motivates our country-level rather than continent-level treatment of policy maturity.

##### Statistical analysis

• Descriptive comparisons: Given the heavy-tailed, nonnormal distribution of the outcome, our descriptive group comparisons relied on distribution-free methods. We used the Mann-Whitney U test,^43^^,^^44^ to assess location shifts and report the rank-biserial correlation as a non-parametric effect size measure.^45^ The magnitude of the location shift was estimated using the Hodges-Lehmann estimator.^46^^,^^47^ Homogeneity of variances was tested using a robust Levene-type test based on medians.^48^^,^^49^ For context, parametric mean differences on the log-transformed outcome with bootstrap confidence intervals are also reported.

##### Predictive modeling

A Random Forest Regressor (Figure 9) was implemented to predict capacity while guarding against overfitting and data leakage. The model was configured with n_estimators = 800, max_depth = None, and min_samples_leaf = 2 (Table 3). Crucially, evaluation was performed using GroupKFold cross-validation (k = 5), grouping by Country (with a fallback to Technology_agg) to prevent inflationary performance estimates from geographic or technological leakage. Model performance was assessed using out-of-fold (OOF) R^2^, Mean Absolute Error (MAE), and Root Mean Squared Error (RMSE) on the log scale (Equation 5).

(Equation 5) $$R^{2}=1-\frac{{\sum_{i}\left(y_{i}-{\hat{y}}_{i}\right)}^{2}}{{\sum_{i}\left(y_{i}-y_{i}\right)}^{2}},MAE=\frac{1}{n}\sum_{i}|y_{i}-{\hat{y}}_{i}|\text{,}RMSE=\sqrt{{\frac{1}{n}\sum_{i}\left(y_{i}-{\hat{y}}_{i}\right)}^{2}}$$

### Quantification and statistical analysis

#### Causal estimation and identification

We estimated the Average Treatment Effect (ATE) under the assumptions of consistency, conditional ignorability (*Y*(0),*Y*(1))⊥*T*|*X*), and positivity. Positivity was enforced via propensity score trimming. The propensity score model was evaluated using the Area Under the Receiver Characteristic Curve (AUC) and the Brier score (Equation 6)^50^ to assess predictive performance and calibration, respectively. The region of common support on the other hand was assessed by comparing the propensity score distributions of treated and control units using the Kolmogorov-Smirnov (KS) distance statistic (Equation 7).^51^ Where *F*_1_ and *F*_0_ are the empirical distribution functions of the propensity scores for the treated and control groups, respectively. Analyses were restricted to the region of common support where *D*_*KS*_ was minimized and absolute standardized mean differences (ASMD) for all confounders were below 0.10.

(Equation 6) $$\text{Brier}=\frac{1}{n}\sum_{i=1}^{n}{\left(T_{i}-\hat{e}\left(X_{i}\right)\right)}^{2}$$

(Equation 7) $$D_{KS}=\sup\left|F_{1}\left(x\right)-F_{0}\left(x\right)\right|$$

We triangulated evidence using three distinct groups of estimators (Table 4) to enhance robustness.

• Adjusted Means (back-door adjustment) defined in Equation 8.

(Equation 8) $${\hat{ATE}}_{ADM}=\frac{1}{n}\sum_{i=1}^{n}\left[\hat{m}\left(1,X_{i}\right)-\hat{m}\left(0,X_{i}\right)\right],\hat{m}\left(t,X_{i}\right)=E\left[Y\;|\;T=t,X\right]$$

Where $\hat{m}\left(t,X_{i}\right)$ is the predicted outcome from linear model.

• Inverse Probability Weighting (IPW) as defined by Equation 9.

(Equation 9) $${\hat{ATE}}_{IPW}=\frac{\sum_{i}\left(\frac{T_{i}Y_{i}}{\hat{e}\left(X_{i}\right)}-\frac{\left({1-T}_{i}\right)Y_{i}}{1-\hat{e}\left(X_{i}\right)}\right)1\left\{\hat{e}\left(X_{i}\right)\in \left[\alpha ,1-\alpha \right]\right\}}{\sum_{i}1\left\{\hat{e}\left(X_{i}\right)\in \left[\alpha ,1-\alpha \right]\right\}}$$

Where $\hat{e}\left(X_{i}\right)$ is the estimated propensity score from a regularized logistic regression.

• Doubly Robust (DR) Estimation (Equation 10).

(Equation 10) $${\hat{ATE}}_{DR}=\frac{1}{n}\sum_{i=1}^{n}\left[\left(\frac{T_{i}}{\hat{e}\left(X_{i}\right)}-\frac{\left({1-T}_{i}\right)}{1-\hat{e}\left(X_{i}\right)}\right)\left(Y_{i}-\hat{m}\left(T_{i},X_{i}\right)\right)+\hat{m}\left(1,X_{i}\right)-\hat{m}\left(0,X_{i}\right)\right]$$

Effect estimates on the log scale were back-transformed to capacity ratios (Equation 11).

(Equation 11) $$Capacity\;ratio=10^{\Delta }$$

• Uncertainty Quantification and Multiple Testing: Uncertainty for all point estimates (e.g., ATE, HTE, location shifts) was quantified using nonparametric bootstrapping, with resamples to ensure stable estimates, generating percentile-based confidence intervals as depicted in Equation 12. To control the false discovery rate across the multiple hypotheses tested in our subgroup (HTE) analyses, all reported p-values for HTE are adjusted unless otherwise stated using the Benjamini-Hochberg procedure (see below).^52^ *B* = 200(1-*α*)

(Equation 12) $${CI}_{1-\alpha }=\left[q_{\alpha /2}\left({\hat{\theta }}^{*}\right),q_{1-\alpha /2}\left({\hat{\theta }}^{*}\right)\;\right]$$

• Multiple Testing Correction: For all pre-registered subgroup (HTE) analyses, we controlled the False Discovery Rate (FDR) at q = 0.10 using the Benjamini-Hochberg procedure. The procedure rejects the null hypothesis for all tests for which Equation 13.^53^ $H_{0,\left(1\right)},\ldots ,H_{0,\left(k\right)}p\left(k\right)\leq \frac{k}{m}q$

(Equation 13) $$p\left(k\right)\leq \frac{k}{m}q\;\Rightarrow \;reject\;H_{0,\left(1\right),\ldots \ldots .,}H_{0,\left(k\right)}$$

#### Moderator analysis (HTE)

Pre-registered strata (scale, region/policy, maturity/status) are re-estimated with within-stratum identification after support/balance checks as in Equation 10. Continuous heterogeneity is profiled via CATEs from meta-learners. Strata failing support or balance gates are labeled exploratory and excluded from headline claims. Minimum sample safeguards (≥50 total; ≥15 treated and ≥15 controls) prevent degenerate fits. Cross-references to Results: Figures 14A–14D and 15A–15D; Tables S3–S7.

#### Robustness and sensitivity

Sensitivity is probed across propensity specifications, outcome transforms ($\log_{10},\log\left(.+1\right),\sqrt{.}$), trimming bands, and matching variants (radius, kernel, caliper). Uncertainty uses nonparametric bootstraps; when estimator families are compared, conservative multiplicity control is applied. A Rosenbaum-style analysis reports the *Γ* at which qualitative conclusions would flip. Re-estimating within maturity/status bands mitigates announcement bias.

#### Software and reproducibility

All analyses were performed in Python 3.10 using standard scientific libraries (Pandas, NumPy, SciPy, Scikit-learn) and specialized causal inference packages (EconML, DoWhy). The computational environment was managed via a virtual environment with explicitly pinned dependencies to ensure full reproducibility. All random processes were seeded (seed = 42). The complete analytical code is publicly available at https://github.com/NkJoseph/CCUS-impact-H2-Scalability and the harmonised analysis set is deposited at Mendeley Data: https://doi.org/10.17632/cjdm993dnn.1 see resource availability for the full list of deposits.
