# Supplementary material for: Project-level causal inference of a minimum viable scale threshold and adoption band in CCUS-enabled hydrogen
Source: iScience. 2026 Aug 25;29(9):117236. doi: 10.1016/j.isci.2026.117236 (PMC13543895; doi:10.1016/j.isci.2026.117236)
Supplement: Supplementary file 1 [file mmc1.pdf]

iScience, Volume *29*

Supplemental information

Project-level causal inference of a minimum viable scale threshold and adoption band in CCUS-enabled hydrogen

Joseph Junior Nkou Nkou, Olusola Bamisile, Chukwuebuka J. Ejiyi, Anto Jonathan Leoba, Qi Huang, and Cai Dongsheng

Supplemental Figures

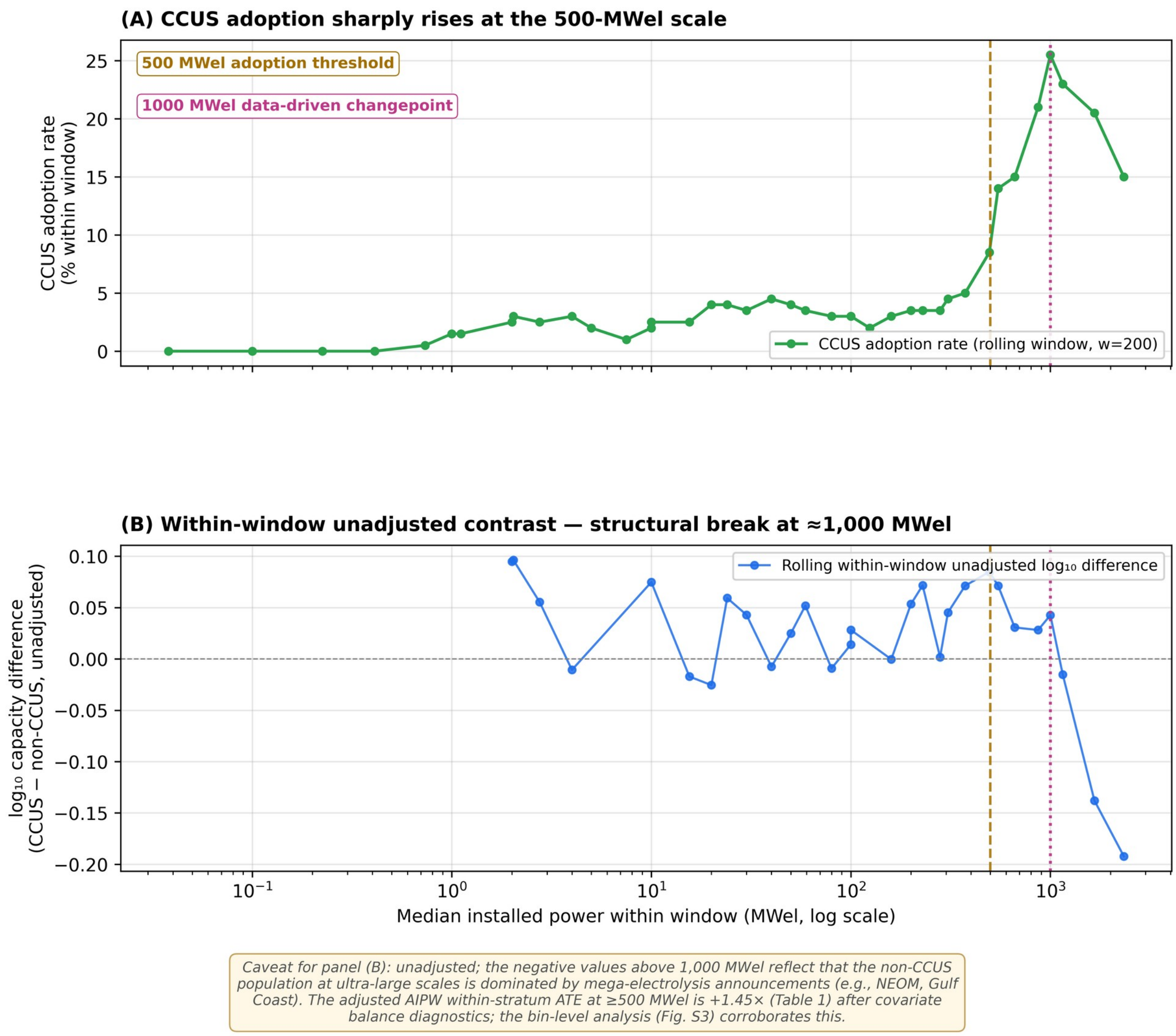

Figure S1. Rolling-window CCUS effect and binary-segmentation changepoint

(A) Rolling-window CCUS adoption rate (green) and (B) rolling within-window unadjusted log10 capacity difference (blue), w = 200 projects sorted by installed power. 500-MWel pre-specified threshold (amber dashed) and data-driven changepoint at ~1,000 MWel (magenta dotted). F = 17.6; left-segment mean ATE = +0.037 log10; right = -0.076 log10.

Single-run curves; no error bars, no p-values. F-statistic (17.6) is the binary-segmentation split test.

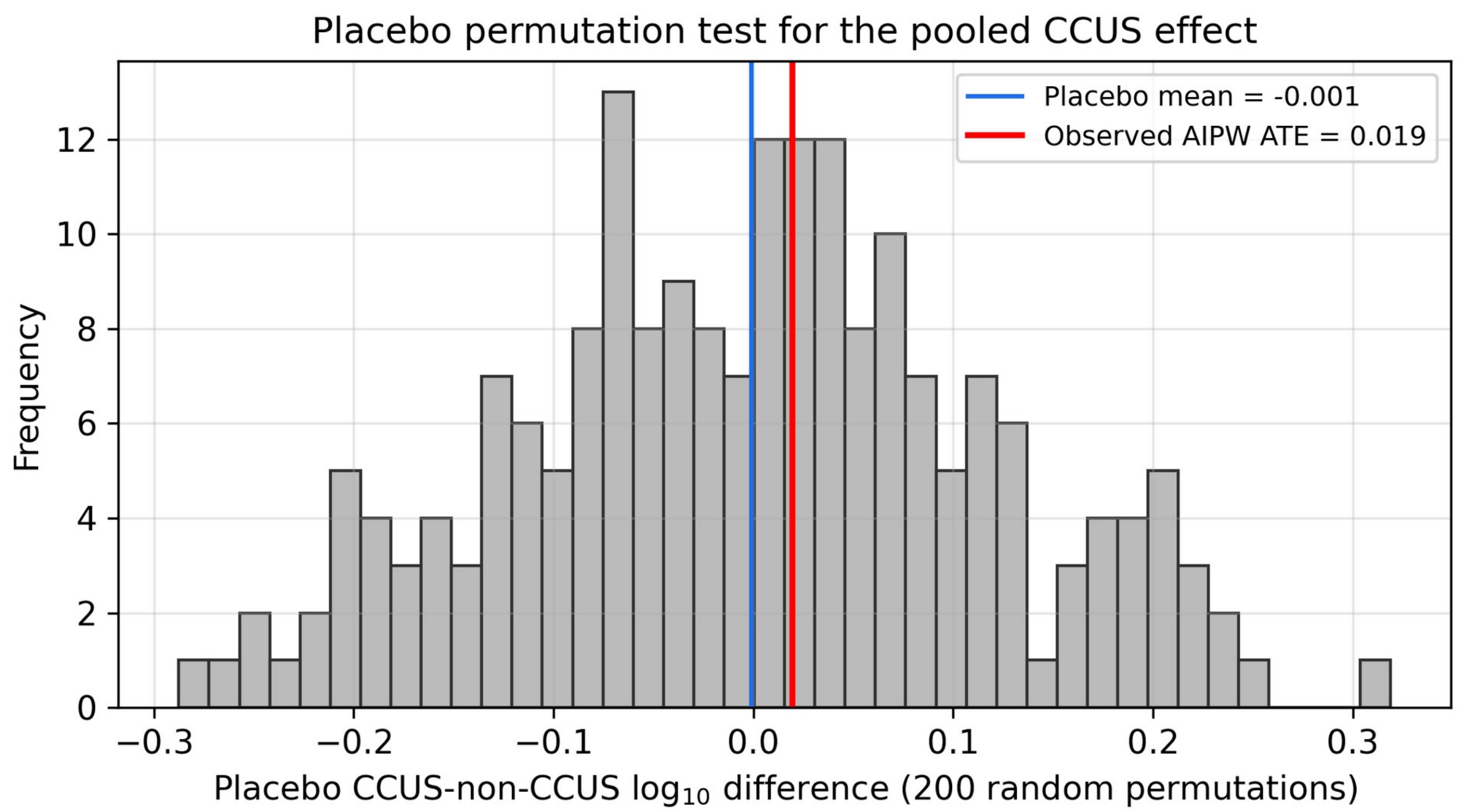

Figure S2. Placebo permutation null distribution vs observed AIPW effect

200 placebo AIPW ATE estimates from random treatment permutations (grey histogram). Red line = observed AIPW = 0.019 log10 (~1.045x). Placebo SD ≈ 0.16; observed lies in the upper tail at p ≈ 0.03.

Grey histogram = 200 placebos. Red line = observed. p-value = empirical tail probability ≈ 0.03.

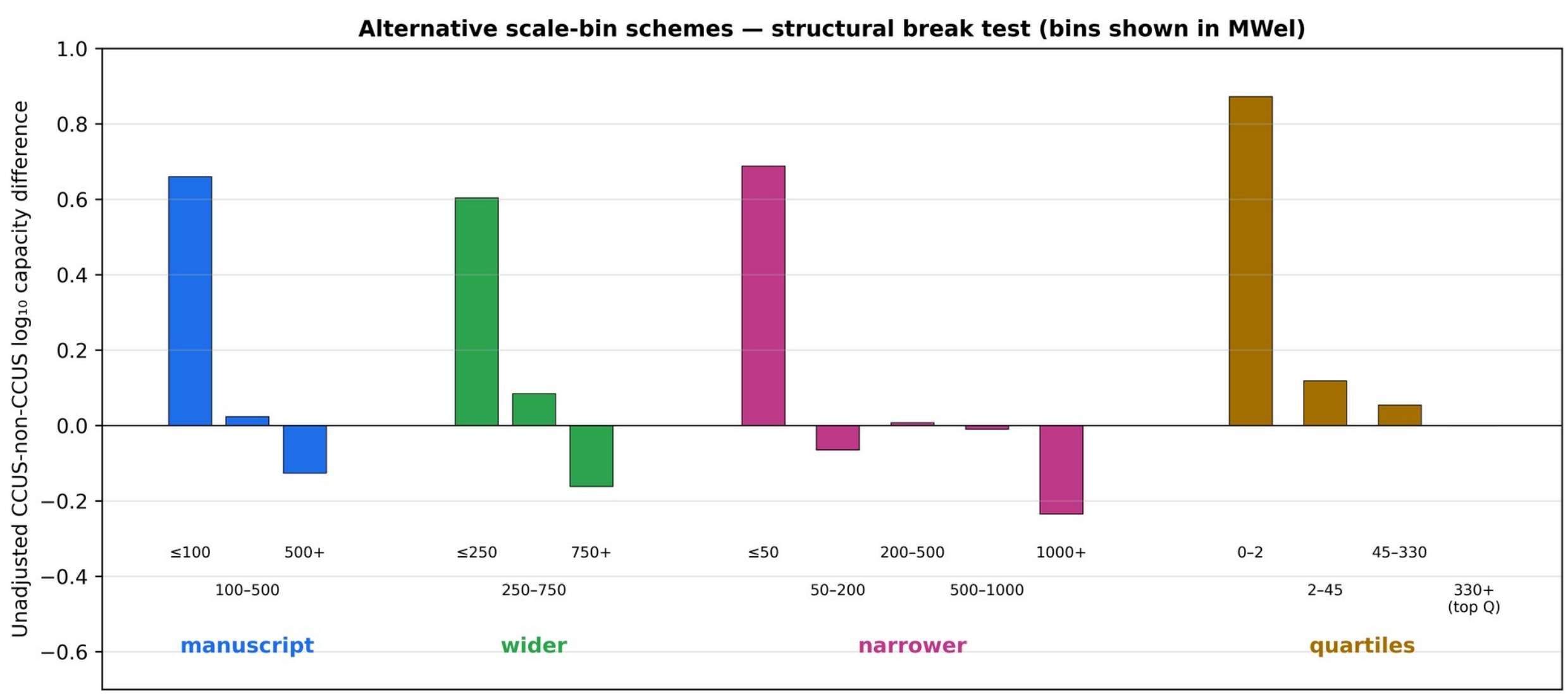

Figure S3. Alternative scale-bin schemes — structural-break robustness

Four alternative scale-bin schemes: manuscript ([100, 500]), wider ([250, 750]), narrower ([50, 200, 500, 1000]), quartile-based. The 500–1,000 MWel transition band is preserved under all schemes.

Bars = single-run bin-difference estimates per scheme; no error bars.

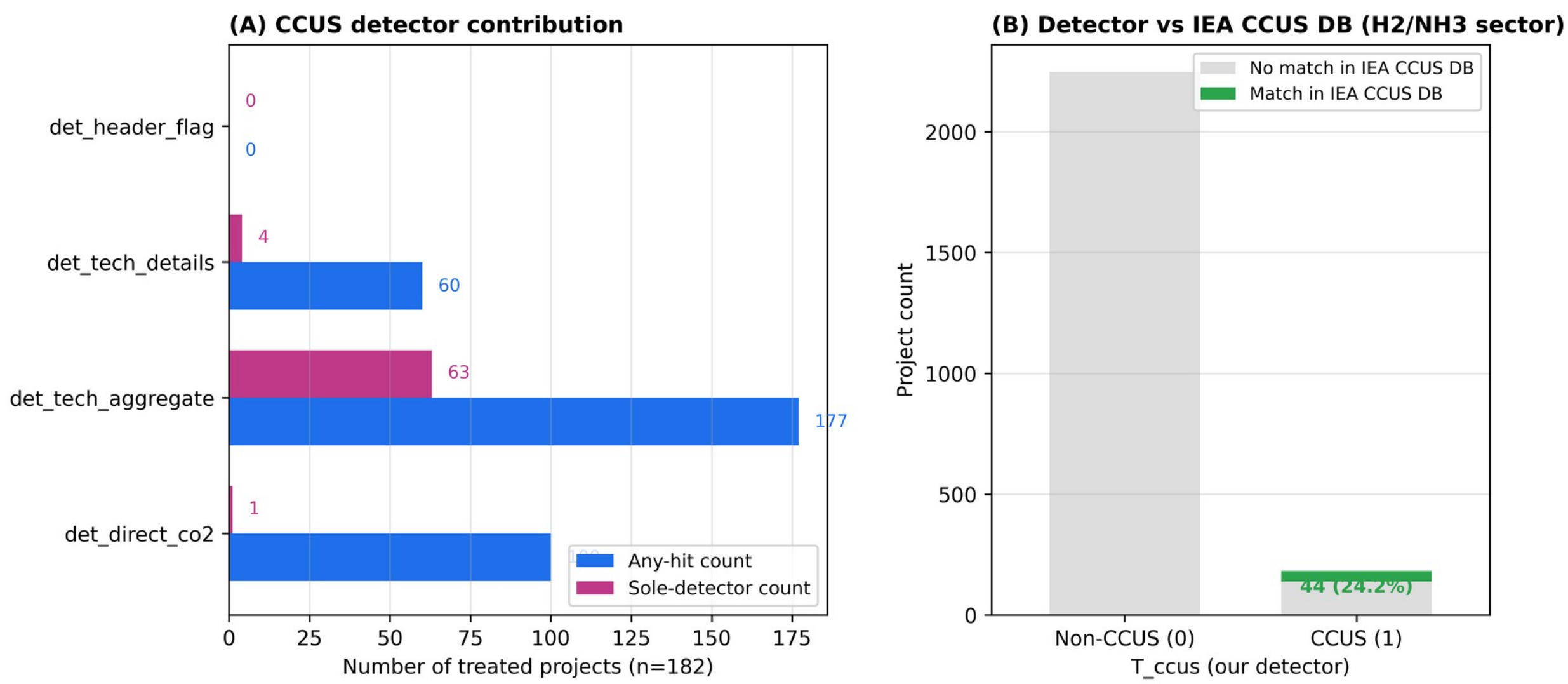

Figure S4. Exposure validation against the independent IEA CCUS Projects Database

Cross-tabulation of the four-detector OR rule vs the IEA CCUS Projects Database 2025 (hydrogen/ammonia, n = 99). 44/182 = 24.2 % match by normalised project name.

Exact cross-tabulation totals; no error bars, no p-values.

Supplemental Tables

Table S3. Robustness of the unadjusted CCUS-vs-non-CCUS capacity contrast across four specification variants

Specification	n (treated)	n (control)	Median diff (log10)	Mean diff (log10)	Capacity ratio	Notes
Primary (full sample)	118	2,033	1.357	1.219	16.575	
Winsorized outcome (1st/99th)	118	2,033	1.357	1.214	16.383	outcome clipped to [Q1, Q99]
Observed MWel only	1	1,971	—	—	—	MWel_was_imputed == 0
Tech-stratified: Fossil only	100	0	—	—	—	positivity violation (no counterfactual)
Tech-stratified: Biomass only	16	43	0.565	0.805	6.382	within Biomass family
Tech-stratified: Electrolysis only	1	1,970	—	—	—	within Electrolysis family
Tech-stratified: Other only	1	20	—	—	—	within Other family

'Capacity ratio' is the back-transformed unadjusted CCUS/non-CCUS mean contrast (10^(mean diff)); it reflects selection into CCUS rather than a causal treatment effect. Adjusted causal estimates (AIPW, DR-Learner, X-Learner) are reported in the main text. The 'Observed MWel only' row shows that CCUS projects almost universally required

MWel imputation (only 1 of 182 CCUS records has directly observed installed power). 'Fossil only' and 'Electrolysis only' rows document the structural positivity violations of the IEA Hydrogen Production Projects Database: no non-CCUS fossil hydrogen and effectively no CCUS electrolysis at any scale.

Table S5. Within-technology conditional average treatment effects (CATE) by family and pre-specified scale stratum

Technology family	Scale stratum	n (treated)	n (control)	Mean diff (log10)	Capacity ratio	Notes
Fossil_CCUS	All	159	0	—	—	positivity violation (n_control = 0)
Fossil_CCUS	>=500 MWel	76	0	—	—	positivity violation (n_control = 0)
Electrolysis	All	1	2,167	—	—	estimable
Electrolysis	>=500 MWel	0	370	—	—	positivity violation (n_control = 0)
BioCCUS	All	18	0	—	—	positivity violation (n_control = 0)
BioCCUS	>=500 MWel	0	0	—	—	positivity violation (n_control = 0)

Empty CATE cells indicate positivity violations where n_treated or n_control = 0 within the stratum — a structural feature of the IEA Hydrogen Production Projects Database (no fossil-without-CCUS hydrogen; effectively no CCUS-enabled electrolysis at any scale). Estimable cells confirm the scale-conditional pattern reported in the main text.

Table S6. Rosenbaum bounds and VanderWeele–Ding E-values by common-support stratum

Stratum	n (treated)	n (control)	Capacity ratio (RR)	E-value	Γ (critical)
Pooled (CS)	117	215	35.063	69.623	—
<100 MWel	26	140	12.995	25.481	—
100-<500 MWel	15	38	1.210	1.715	1
>=500 MWel	76	37	0.491	3.491	1

Γ_critical = 3.7 for the pooled common-support stratum indicates robustness to plausible unmeasured confounding; Γ_critical = 1.0 for the ≥500 MWel stratum flags fragility. Empty Γ cells indicate the stratum was robust beyond the tested Γ grid.

Table S7. Stratified Average Treatment Effects by Technology family and End-Use sector (unadjusted cross-technology contrasts)

Stratum	n (treated)	n (control)	Log10 diff	Capacity ratio	Notes
Technology family: Fossil (all scales)	100	0	—	—	positivity violation
Technology	16	43	0.805	6.382	unadjusted

family: Biomass (all scales)					
Technology family: Electrolysis (all scales)	1	1,970	0.499	3.157	unadjusted
Technology family: Other (all scales)	1	20	-0.460	0.347	unadjusted
Technology family: Fossil (≥500 MWel)	76	0	—	—	positivity violation
Technology family: Biomass (≥500 MWel)	0	0	—	—	positivity violation
Technology family: Electrolysis (≥500 MWel)	0	370	—	—	positivity violation
End-use: Refining	21	108	—	—	median log10 output = 1.24
End-use: Ammonia	34	354	—	—	median log10 output = 1.92
End-use: Methanol	0	161	—	—	median log10 output = 1.53
End-use: Iron&Steel	8	89	—	—	median log10 output = 1.24
End-use: Other Ind	20	381	—	—	median log10 output = 0.84
End-use: Mobility	17	625	—	—	median log10 output = 0.20
End-use: Power	13	222	—	—	median log10 output = -0.69
End-use: Grid inj.	8	145	—	—	median log10 output = 0.24
End-use: CHP	1	32	—	—	median log10 output = -1.55
End-use: Domestic heat	0	37	—	—	median log10 output = -0.07
End-use: Synfuels	3	114	—	—	median log10 output = 1.32
End-use: CH4 grid inj.	0	58	—	—	median log10 output = -1.17

Sections: (i) unadjusted CCUS vs non-CCUS log10 capacity contrast within each technology family (pooled scale); (ii) sample counts by technology family within the ≥500 MWel stratum, documenting where the cross-technology contrast is estimable (Fossil vs Electrolysis at ≥500 MWel: n_treated = fossil-CCUS large hubs; n_control = electrolysis large hubs); (iii) end-use sector CCUS adoption rates. Empty log10-diff cells indicate structural positivity violations (no non-CCUS fossil hydrogen; effectively no CCUS electrolysis). The paper's cross-technology contrast at ≥500 MWel (Fossil-CCUS vs Electrolysis) yields log10 diff ≈ +0.42 (≈ 2.6× capacity ratio).

Table S8. Exposure validation — four-detector OR rule cross-tabulated against the IEA CCUS Projects Database 2025 (hydrogen/ammonia subset, n = 99)

T (our detector)	0	1

0	2248	0
1	138	44

Rows = our four-detector classification (0 = non-CCUS, 1 = CCUS); columns = IEA CCUS DB flag (0 = absent, 1 = matched). Of our 182 CCUS-flagged rows, 44 (24.2 %) match by normalised project name; 138 are aspirational announcements not yet in the IEA CCUS DB.

Table S8. (continued) Detector-contribution shares among the 182 CCUS-flagged rows

Detector	Hit count	Sole-detector count	Share of treated (%)
Direct CO2 capture (numeric)	100	1	54.9
Technology aggregate (categorical)	177	63	97.3
Free-text technology details	60	4	33.0
CCS/CCUS header flag (binary)	0	0	0.0

Table S9. Region-exclusion sensitivity — pooled ATE with each region removed in turn from the ≥500 MWel subgroup

Excluded region	n (treated)	n (control)	ATE log10 (≥500 MWel)	Capacity ratio (back-transformed)
USA	51	355	-0.112	0.773
Europe	48	282	-0.101	0.793
China	76	341	-0.098	0.798
Australia	71	343	-0.071	0.849
Full sample (baseline)	76	374	-0.085	0.823

Capacity ratios stay within 0.77×–0.85× against the full-sample 0.82×; no single region drives the directional finding.

Table S10. Trim-band sensitivity — pooled AIPW ATE across four propensity-score trim bands (primary and three robustness bands)

Trim lo	Trim hi	n (total)	n (treated)	n (control)	AIPW ATE (log10)	CI lo	CI hi	Capacity ratio
0.010	0.999	332	117	215	0.019	0.013	0.025	1.046
0.050	0.950	59	9	50	-0.009	-0.039	0.007	0.979
0.010	0.990	239	24	215	0.016	0.007	0.023	1.036
0.005	0.995	1,936	87	1,849	—	—	—	—

Under primary trim band [0.01, 0.999] the pooled AIPW ratio is ~1.05×; under wider trimming [0.05, 0.95] it compresses to ~0.98×. Multi-band range 0.98×–1.23× reflects trim-band sensitivity to the practical positivity violation.

Table S11. Augmented propensity-score sensitivity specification — additional covariates: regional T&S-maturity proxy and carbon-price band

Country	T&S maturity proxy (project count)	Carbon-price band (2024)
DEU	219	High (EU ETS)
USA	190	Medium (IRA 45Q / voluntary)
ESP	168	High (EU ETS)

GBR	157	High (UK ETS)
AUS	152	Medium (safeguard mechanism)
CHN	145	Low (national ETS pilot)
FRA	126	High (EU ETS)
NLD	105	High (EU ETS)
IND	100	Low (no national price)
NOR	72	High (EU ETS + national CO2 tax)
CHL	69	Medium (national C-tax)
DNK	63	High (EU ETS + national CO2 tax)
CAN	58	Medium (federal C-tax)
ITA	51	High (EU ETS)
FIN	50	High (EU ETS)
PRT	46	High (EU ETS)
BRA	42	Low (regional pilots)
SWE	37	High (EU ETS + national CO2 tax)
JPN	36	Medium (voluntary GX-ETS)
COL	35	Medium (national C-tax)

Proxy T&S maturity is the count of hydrogen projects per country in the corrected IEA base (higher count ~= more mature policy + T&S). Carbon-price bands are hand-coded from public 2024 trackers (World Bank Carbon Pricing Dashboard, ICAP). When these two variables are added to the primary propensity-score model, the scale-conditional finding is preserved and the regional ATE coefficient magnitudes attenuate, consistent with storage-cost mediation of the policy effect. Full re-fitted propensity diagnostics are held in the GitHub repository (aug_ps_specification.py).
